# Nano-Enabled Advances in Tea Tree Essential Oil (*Melaleuca alternifolia*): Composition, Bioactivity, and Emerging Roles in Food Protection

**DOI:** 10.3390/ma19132915

**Published:** 2026-07-07

**Authors:** Huy Loc Nguyen, Hong Minh Xuan Nguyen, Thi Bich Ngoc Nguyen

**Affiliations:** 1 Department of Engineering and Technology, Van Hien University, Ho Chi Minh City 72508, Vietnam; 2 Department of Chemical Engineering and Food Technology, Nong Lam University, Ho Chi Minh City 71308, Vietnam; nmxhong@hcmuaf.edu.vn; 3 Department of Water Management and Hydrological Sciences, Texas A&M University, College Station, TX 77843-2117, USA

**Keywords:** tea tree oil, terpinen-4-ol, antimicrobial, nanoemulsions, encapsulation, food safety

## Abstract

Tea tree essential oil (TTO), extracted from *Melaleuca alternifolia*, is a terpene-rich botanical antimicrobial with demonstrated broad-spectrum activity against foodborne pathogens and spoilage microorganisms. Its bioactivity is principally attributed to oxygenated monoterpenes, most notably including terpinen-4-ol, γ-terpinene, and α-terpinene, whose structure–activity relationships govern interactions with microbial membranes and intracellular targets. This review provides a comprehensive, mechanistically grounded analysis of TTO as a sustainable antimicrobial platform for food preservation applications. The physicochemical determinants of TTO performance are critically assessed, encompassing chemotype-dependent compositional variability, hydrophobicity, limited aqueous solubility, and oxidative instability, with emphasis on how these properties constrain efficacy in complex food matrices. Antimicrobial mechanisms are systematically examined, including membrane permeabilization, disruption of cellular homeostasis, oxidative stress induction, and quorum-sensing interference. Focus is placed on nanostructured delivery systems, including nanoemulsions, biopolymer-based encapsulants, and hybrid nanocomposites, that improve physicochemical stability, modulate release kinetics, and potentiate antimicrobial activity. The integration of these engineered formulations into edible coatings, active packaging, and sanitation protocols across fresh produce, meat, and dairy systems is evaluated in the context of practical food safety applications. Translational limitations are addressed, including volatility, sensory incompatibility, regulatory constraints, and concentration-dependent cytotoxicity considerations. Collectively, this review positions TTO-based nanoformulations as a scientifically promising and technologically scalable approach to next-generation food preservation, while identifying critical gaps that must be resolved to support regulatory acceptance and commercial implementation.

## 1. Introduction

Tea tree essential oil (TTO) has emerged as a promising natural antimicrobial for food preservation. However, its translation into practical food systems remains limited by poor aqueous dispersibility, volatility, and oxidative instability. These formulation challenges have accelerated the development of nano-enabled delivery systems designed to improve the stability, bioavailability, and antimicrobial efficacy of TTO (Table 1).

The rising consumer demand for natural, clean-label, and minimally processed foods has heightened research interest in plant-derived essential oils as alternatives to synthetic preservatives [3]. TTO has garnered significant attention due to its potent antibacterial, antifungal, antiviral, and antioxidant activities, mostly attributed to its terpinen-4-ol-rich composition [4]. The global food business faces mounting issues related to microbial contamination, resistance to chemical preservatives, and the persistence of foodborne pathogens on food and food-contact surfaces. TTO has been extensively investigated for applications including fresh produce sanitation, edible coatings, packaging films, and surface cleaning technologies [5,6]. This paper offers a thorough examination of TTO’s physicochemical characteristics, antibacterial mechanisms, and novel uses in food safety.

Tea tree essential oil comprises a complex amalgamation of over 100 elements, predominantly monoterpenes, sesquiterpenes, and their respective alcohols, with terpinen-4-ol, γ-terpinene, α-terpinene, and 1,8-cineole being the principal ingredients [7]. The chemical composition of TTO is affected by factors like plant genotype, geographic origin, climatic circumstances, and extraction process, leading to diversity in biological activity [8]. The physicochemical properties of TTO, namely hydrophobicity, volatility, solubility behavior, and oxidative stability, directly affect its antibacterial efficacy and dictate its appropriateness for integration into food matrices [9]. The compound’s restricted water solubility limits its direct use in hydrophilic foods, requiring encapsulation methods or emulsification procedures to improve dispersion and provide controlled release. Comprehending these inherent features is essential for developing robust, efficient delivery methods for food applications.

The antibacterial efficacy of TTO has been extensively recorded against prominent foodborne pathogens, such as *Listeria monocytogenes*, *Escherichia coli*, *Salmonella enterica*, *Staphylococcus aureus*, *Campylobacter jejuni*, and several spoilage fungi [10,11]. The mechanism of action is mainly due to its capacity to infiltrate and damage microbial cell membranes, enhancing permeability and resulting in the release of internal constituents.

Tea tree oil (TTO) differs fundamentally from engineered nanoparticles in both structure and mode of antimicrobial action yet shares functional similarities in food and environmental applications. As a complex mixture of volatile terpenes, TTO exerts antimicrobial effects primarily through membrane disruption, increased permeability, and interference with cellular metabolism. In contrast, nanoparticles such as silver nanoparticles (AgNPs), zinc oxide (ZnO), or metal–organic frameworks (e.g., ZIF-8) rely on mechanisms including reactive oxygen species (ROS) generation, ion release, and surface-mediated interactions [12,13]. Compared to inorganic nanoparticles, TTO offers advantages in terms of biodegradability, lower toxicity, and partial regulatory acceptance in non-food or indirect applications, with limitations for direct food use in the United States. However, it suffers from limitations such as volatility, poor water solubility, and instability under oxidative conditions. Poor aqueous solubility represents one of the major barriers preventing direct application of TTO in aqueous foods. To improve dispersion, nanoemulsions, liposomes, polymeric nanoparticles, and lipid-based nanocarriers have been extensively developed to improve dispersion, increase interfacial contact with microorganisms, and enhance antimicrobial efficacy while reducing the required oil concentration. Nanoparticles, on the other hand, provide higher structural stability, tunable surface properties, and controlled release capabilities. Increasingly, hybrid systems that encapsulate TTO within nanocarriers combine the natural bioactivity of essential oils with the stability and delivery efficiency of nanomaterials, representing a promising direction for advanced antimicrobial applications.

Terpinen-4-ol, the principal active chemical, engages with membrane phospholipids, resulting in structural destabilization, a reduction in proton motive force, metabolic inhibition, and ultimately cell death [14]. Alternative antimicrobial mechanisms, including oxidative stress induction, enzyme inhibition, and disruption of quorum sensing, have also been suggested [15]. These multi-target processes diminish the probability of microbial resistance in comparison to traditional preservatives. TTO demonstrates significant anti-biofilm efficacy by inhibiting cell adhesion, dissolving pre-existing biofilms, and modifying the integrity of extracellular polymeric substances (EPS), rendering it especially pertinent for the cleanliness of food-contact surfaces and the management of biofilm-related contamination [16].

**Figure 1:**
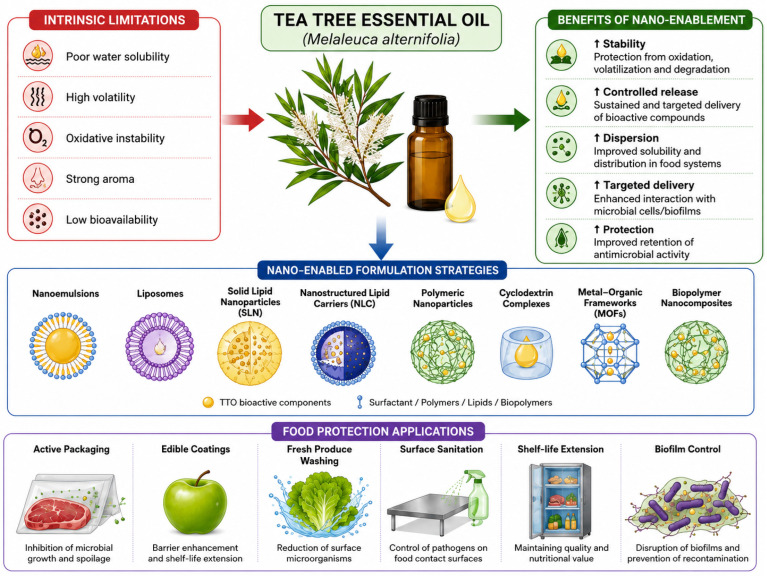
Conceptual overview of nano-enabled strategies for improving the stability, delivery, and food protection performance of tea tree essential oil (created by BioRender (Biorender.com) and modified by Figure Labs (Figurelabs.ai)). The left panel summarizes the intrinsic limitations of TTO, including poor aqueous solubility, high volatility, oxidative instability, strong aroma, and low bioavailability. The center panel presents TTO as the active antimicrobial agent. The right panel highlights the major benefits provided by nano-enabled formulations, including enhanced stability, controlled release, improved dispersibility, targeted antimicrobial delivery, and prolonged protection. The lower panel summarizes representative nano-enabled delivery platforms (nanoemulsions, liposomes, solid lipid nanoparticles, nanostructured lipid carriers, polymeric nanoparticles, cyclodextrin complexes, metal–organic frameworks, and biopolymer nanocomposites) together with their major food applications, including active packaging, edible coatings, produce washing, surface sanitation, shelf-life extension, and biofilm control. The corresponding topics are discussed in Section 2, Section 3, Section 4, Section 5 and Section 6.

Increasing evidence endorses the prospective incorporation of TTO into food safety measures. TTO-containing wash solutions and vapor-phase treatments have shown substantial reductions in bacteria and spoilage fungi on leafy greens, berries, and ready-to-eat goods in fresh produce systems [17]. TTO-infused edible coatings, often composed of biopolymers like chitosan, alginate, or whey protein isolate, establish antimicrobial barriers that prolong shelf life while preserving sensory qualities [18]. The integration of biodegradable packaging films, through direct blending or nanoencapsulation, has demonstrated potential in improving mechanical characteristics, facilitating prolonged antimicrobial release, and mitigating microbial proliferation on perishable commodities such as meat and cheese [19]. Furthermore, encapsulated TTO nanoparticles, including liposomes, nanoemulsions, solid lipid nanoparticles, and cyclodextrin inclusion complexes, have been engineered to mitigate volatility and enhance stability, controlled release, and compatibility with food matrices [20]. These advancements underscore the adaptability of TTO within novel nanotechnology-based food preservation systems (Figure 1).

Figure 1 summarizes the overall framework of this review by linking the intrinsic limitations of TTO with nano-enabled formulation strategies, their resulting functional advantages, and representative food protection applications. The following sections discuss each of these components in detail, beginning with the physicochemical properties of TTO, followed by antimicrobial mechanisms, nano-enabled delivery systems, practical food applications, and future challenges.

Notwithstanding its robust antibacterial activity, certain difficulties impede the extensive utilization of TTO in food systems. The strong scent and chemical instability when exposed to light and oxygen at elevated concentrations require meticulous formulation and dosage optimization. However, the current literature rarely establishes application-specific safe dosage thresholds, particularly distinguishing between direct food incorporation and indirect exposure via active packaging systems, resulting in uncertainty in practical risk assessment [21]. Moreover, interactions with intricate food matrices can diminish antibacterial efficacy due to binding with proteins, lipids, and polysaccharides [22]. Regulatory factors also affect its adoption, as regulatory approval remains limited and application-dependent; notably, tea tree oil is not classified as Generally Recognized as Safe (GRAS) for direct food use in the United States, despite its inclusion in FEMA flavor ingredient listings, which do not constitute FDA approval. Thus, current research emphasizes enhancing delivery systems, assessing sensory effects, comprehending synergistic antimicrobial interactions with other natural compounds (e.g., carvacrol, thymol, cinnamaldehyde), and determining safety profiles by toxicological and in vivo investigations [23].

Although tea tree essential oil possesses broad-spectrum antimicrobial activity [24], its practical application in food systems remains constrained by poor aqueous solubility, high volatility, oxidative degradation, and rapid loss of bioactive constituents during processing and storage. These limitations have stimulated extensive research into nano-enabled delivery platforms, including nanoemulsions, liposomes, polymeric nanoparticles, solid lipid nanoparticles, cyclodextrin complexes, and metal–organic framework-based carriers, which substantially improve physicochemical stability, controlled release, and antimicrobial performance. Consequently, recent advances in food preservation increasingly emphasize nanotechnology not merely as a delivery strategy but as an enabling platform for translating the intrinsic bioactivity of TTO into practical food applications. Accordingly, this review integrates the chemistry and antimicrobial mechanisms of TTO with recent developments in nano-enabled formulations, highlighting how nanotechnology bridges fundamental bioactivity and real-world implementation in food protection.

## 2. Physicochemical Properties of Tea Tree Essential Oils

TTO is a complex mixture of volatile phytochemicals, predominantly terpenes and related compounds, which together confer its characteristic physical and chemical properties [25]. Understanding these physicochemical properties, including chemical composition, variability in composition, physical parameters (density, refractive index, etc.), hydrophobicity, volatility, oxidative stability, and interactions with food matrices, is crucial for effectively applying TTO in food safety. These properties influence how TTO can be formulated, delivered, and perform as a natural antimicrobial agent in foods [26]. Moreover, robust analytical methods (e.g., gas chromatography–mass spectrometry and infrared spectroscopy) are used to characterize TTO’s composition and ensure quality and consistency [27]. In this section, we review the physicochemical properties of TTO in detail and discuss how each property impacts potential food safety applications.

### 2.1. Chemical Composition and Variability

**Figure 2:**
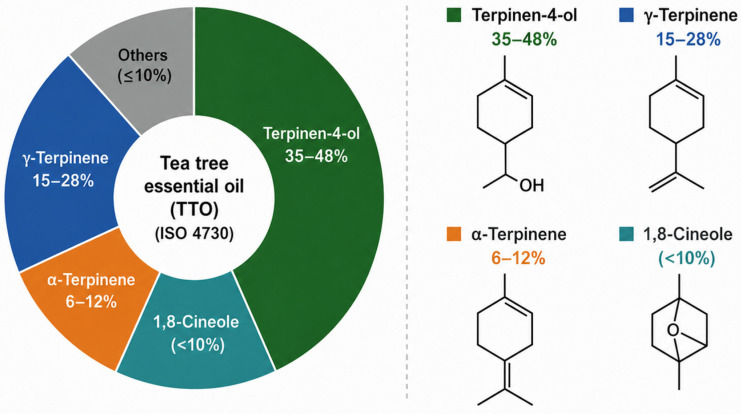
Representative composition of tea tree oil (TTO) based on ISO 4730 standards [29] (created by BioRender and modified by Figure Labs).

Tea tree oil is distilled from the leaves of *Melaleuca alternifolia* and typically contains an assemblage of approximately 100 distinct compounds [28]. Despite this complexity, a relatively small subset of constituents comprises the bulk of the oil. Eight major components, including terpinen-4-ol, γ-terpinene, α-terpinene, 1,8-cineole, terpinolene, p-cymene, α-pinene, and α-terpineol, usually account for about 90% of TTO by mass (Figure 2) [29].

Terpinen-4-ol, a monoterpenol, is the single most abundant component and a key contributor to TTO’s bioactivity. High-quality TTO is defined by a high terpinen-4-ol content (typically 35–48% of the oil) and relatively low content of 1,8-cineole (an oxidized terpene often kept <10%). For example, the international ISO 4730 standard for “Oil of Melaleuca, terpinen-4-ol type” specifies terpinen-4-ol in the range of ~35–48% and limits 1,8-cineole to <10% [30]. Other significant constituents are the monoterpene hydrocarbons (γ-terpinene typically 15–28%, α-terpinene 6–12%, p-cymene up to ~8%) and sesquiterpenes in smaller amounts (e.g., bicyclic sesquiterpenes like aromadendrene, viridiflorene, and cadinenes each usually <3%). This overall composition gives TTO a clear, colorless to pale yellow appearance and a strong medicinal camphoraceous aroma (Table 2).

The principal constituents of tea tree oil exhibit molecular weights typically ranging from approximately 136 g/mol (e.g., *p*-cymene) to higher values associated with terpinen-4-ol and related sesquiterpenols [32]. Structurally, these compounds comprise aliphatic cyclic hydrocarbons as well as oxygen-containing alcohols. Terpinen-4-ol and α-terpineol are classified as oxygenated terpenes (terpenoids), whereas α-terpinene, γ-terpinene, *p*-cymene, and α-pinene are non-oxygenated hydrocarbon terpenes (Table 3) [33].

The presence of this mix of functional groups (alcohols, alkenes) influences properties like polarity and reactivity. From a food safety perspective, the chemical composition is paramount because the antimicrobial efficacy of TTO is largely attributed to its major constituents, especially terpinen-4-ol, and their synergistic effects. For instance, terpinen-4-ol is known to exhibit strong antimicrobial activity against a range of foodborne pathogens and spoilage organisms, so oils with higher terpinen-4-ol content tend to be more potent antimicrobials [34]. Conversely, 1,8-cineole (eucalyptol) has comparatively weaker antimicrobial action. Thus, excessive cineole content can dilute efficacy while also imparting a harsher aroma/flavor to foods. Ensuring a proper composition (high terpinen-4-ol, low cineole) is therefore critical for food applications, both to maximize antimicrobial performance and to meet quality standards.

Beyond determining antimicrobial activity, the physicochemical characteristics of the major TTO constituents also influence nanoformulation design. Differences in polarity, hydrophobicity, volatility, and oxidative susceptibility affect encapsulation efficiency, carrier selection, release behavior, and overall formulation stability. Table 4 summarizes the relationships between the principal TTO components and representative nano-enabled delivery strategies.

Although the antimicrobial activity of tea tree essential oil is generally attributed to the synergistic action of its terpene constituents, these compounds exhibit distinct physicochemical properties that influence nanoformulation design. Oxygenated terpenes, particularly terpinen-4-ol, possess greater polarity than hydrocarbon monoterpenes and therefore display different encapsulation behavior, release kinetics, and interactions with carrier materials. In contrast, highly hydrophobic constituents such as γ-terpinene and α-terpinene are more susceptible to oxidative degradation and premature volatilization, making lipid-based nanocarriers, polymeric nanoparticles, and cyclodextrin inclusion systems particularly attractive. The chemical composition of TTO should be considered not only in terms of antimicrobial potency but also as an important determinant for selecting appropriate nano-enabled delivery platforms capable of maximizing stability and preserving bioactivity.

The chemical profile of tea tree oil can vary due to genetic and environmental factors, though commercial TTO is relatively standardized by producers. Different chemotypes of *M. alternifolia* exist, yielding oils with distinct dominant compounds. Six chemotypes have been described (one terpinen-4-ol type, one terpinolene type, and four cineole-rich types), but the terpinen-4-ol chemotype is typically cultivated for commercial TTO [35]. Within the terpinen-4-ol type, natural variation still occurs. Lee et al. (2002) documented significant geographic variation in terpene profiles among 615 *M. alternifolia* trees: terpinen-4-ol ranged roughly from 20 to 40% and 1,8–cineole from 1 to 15% depending on location and genetic lineage [36]. The chemical composition of TTO not only determines its antimicrobial activity but also influences the selection of appropriate nanocarriers. Hydrophobic monoterpenes such as terpinen-4-ol and γ-terpinene exhibit limited aqueous dispersibility and susceptibility to volatilization, making encapsulation strategies essential for maintaining their bioavailability during food processing and storage. Understanding TTO composition provides the molecular basis for rational nanoformulation design.

### 2.2. Physical Characteristics

The bulk physical properties of tea tree oil are thoroughly described and contribute to quality control and formulation for culinary applications. Fresh TTO is a transparent, fluid liquid that ranges from colorless to pale yellow, emitting a potent, sharp medicinal aroma often characterized as terpenic or camphoraceous [37]. The oil possesses low viscosity and disperses effortlessly, which is beneficial for coating applications but also implies it can diffuse or evaporate quickly.

The principal measured physical properties of TTO encompass density, specific gravity, refractive index, and optical rotation, among others. High-quality TTO possesses a relative density of approximately 0.885–0.906 at 20 °C, roughly 0.89 g/mL, rendering it less dense than water [7]. This implies that TTO will remain buoyant on water and is likely to create a surface layer unless emulsified.

In culinary applications, its low density and water immiscibility can pose difficulties in attaining uniform dispersion. The refractive index of TTO at 20 °C ranges from 1.475 to 1.482. The refractive index serves as a rapid assessment of purity, while oils exhibiting an out-of-range RI may suggest adulteration or an atypical composition [38]. The refractive index of TTO is notably elevated owing to its dense terpenoid composition, and this optical characteristic may be utilized in situ with refractometry sensors to assess the concentration of TTO in a formulation. The optical rotation of pure tea tree oil generally ranges from +5° to +15°, recorded at 20 °C [39]. This slight dextrorotatory rotation results from the chiral terpene molecules, such as terpinen-4-ol and α-terpineol, in their native enantiomeric excess. Optical rotation is an additional criterion in pharmacopeial monographs and substantial deviations may indicate improper sourcing. For instance, racemic synthetic additions may diminish the overall rotation. Although optical rotation is not directly pertinent to food functionality, it highlights the significance of stereochemistry in natural oils and serves as a method for verifying authenticity [40].

Additional physical constants of TTO include a boiling range of approximately 150 °C to 210 °C due to its composition as a mixture, and it does not vaporize at a singular temperature. Lighter fractions may evaporate around 150 °C, but heavier sesquiterpenes boil above 200 °C. The extensive boiling range indicates the existence of both low molecular weight monoterpenes and higher sesquiterpenes [41]. The flash point of TTO ranges from 56 to 60 °C, categorizing it as a Class III flammable liquid, which presents safety concerns while handling and signifies its considerable volatility. The freezing point of TTO is around –22 °C, indicating that it typically remains in a liquid state at standard freezer conditions [42]. This is advantageous for storage; however, if a TTO-containing product is subjected to freezing (e.g., ice cream or frozen foods), the oil will not crystallize but will instead remain in a liquid state within the frozen matrix, potentially leading to aggregation or migration.

These physical characteristics affect the incorporation of TTO into food systems or packaging. The low density and insolubility in water indicate that, without adequate emulsification, TTO will segregate and accumulate on surfaces or at the liquid-air contact, perhaps resulting in inconsistent antimicrobial activity or pronounced localized flavor [43]. The volatility indicates that TTO may be lost from open systems, although it also implies that it can function as an active antibacterial in the vapor phase within food packaging headspace. Finally, assessing parameters such as density and refractive index can confirm that a TTO batch complies with specifications prior to its incorporation into a food formulation and any discrepancies may suggest degradation or adulteration that could compromise its efficacy or safety.

In quality control for food applications, producers often verify that the specific gravity and refractive index of incoming TTO ingredients fall within standard ranges as a quick confirmation of purity. For instance, an unusually high refractive index or density might suggest contamination with less-volatile residues or carrier oils [44]. By ensuring these physical constants are correct, manufacturers can be more confident that the TTO’s composition is correct and thus will perform as expected in terms of antimicrobial activity and sensory impact.

### 2.3. Hydrophobicity and Solubility

Tea tree oil is highly hydrophobic, which profoundly affects how it can be used in food systems. From a nanotechnology perspective, this limitation has driven the development of nanoemulsions, lipid nanoparticles, polymeric nanocarriers, and inclusion complexes that increase aqueous dispersibility while simultaneously enhancing antimicrobial contact with microorganisms. Therefore, hydrophobicity should be regarded not only as a physicochemical limitation but also as the primary rationale for nano-enabled formulation design. Chemically, the bulk of TTO’s constituents are non-polar terpenes and terpenoids with very low water solubility [45]. The oil is practically insoluble in water, with an estimated aqueous solubility on the order of only 300–350 mg/L at 25 °C (approximately 0.03% *w*/*v*). In practical terms, if TTO is added to water or a water-based food, the vast majority will not dissolve but remain as an oily phase or droplets. Indeed, TTO is immiscible with water and will spontaneously separate, forming a film or globules. Matussek et al. (2022) [46] succeeded in embedding Tea Tree Oil (TTO) into a biopolymer film and droplet system made from chitosan, and by incorporating Gold Nanoparticles (AuNPs) into the chitosan matrix, they achieved a sustained, controlled release of the oil. This hydrophobic character is quantified by the partition coefficients (log P) of TTO’s components, with the major constituents having log P values ranging roughly from 3 to 5, indicating strong lipophilicity. For example, terpinen-4-ol and α-terpineol (which have a hydroxyl group) have log P values around 2.5–3.5, while purely hydrocarbon terpenes like γ-terpinene and α-terpinene are higher (log P ~5.2–5.3) [47]. These values mean the compounds prefer octanol (a proxy for fats/oils) over water by thousands of fold, hence will overwhelmingly partition into any available non-polar phase.

TTO is miscible with most organic solvents and oils. It is readily soluble in ethanol and other moderately polar organics. For instance, pharmacopoeias note TTO should dissolve clearly in 1–2 volumes of 80–90% ethanol [48]. It also dissolves in fats, vegetable oils, and non-polar solvents like hexane. This means TTO can be blended into oil-based food systems (e.g., certain dressings, oil-based coatings) more easily than into aqueous ones. However, even in lipid-rich foods, partitioning behavior must be considered: TTO may preferentially reside in the lipid phase of a multiphase food (such as an emulsion or a high-fat meat product). In cheese or ground meat, for example, the oil might partly absorb into the fat fraction, which could either concentrate its antimicrobial components away from water-based microbial niches or conversely protect the microbes if they are in the aqueous phase.

The hydrophobicity of TTO necessitates formulation strategies for uniform and effective application in food preservation. One common approach is emulsification, creating a fine oil-in-water emulsion using emulsifiers or encapsulating agents. By forming nano- or micro-emulsions, TTO’s droplets can be dispersed throughout an aqueous food matrix, greatly increasing the surface area of oil in contact with microbes and improving its apparent solubility. For instance, researchers Cen et al. (2025) formulated a nanoemulsion of TTO with ultrasonic emulsification and noted that it significantly enhanced the antibacterial efficacy compared to non-emulsified oil [9]. The nanoemulsion prevented the oil from coalescing and slowed down its evaporation and oxidation, thereby maintaining a higher active concentration in the food system over time. In practical terms, such emulsified TTO could be added to salad dressings, marinades, or beverage emulsions to impart antimicrobial benefits uniformly.

Another strategy is encapsulation of TTO in carriers like cyclodextrins, liposomes, or biopolymer particles. Encapsulation can render the oil dispersible in water and modulate its release. For example, TTO has been incorporated into chitosan nanoparticles and starch-based microcapsules, which remain dispersed in aqueous solutions and then slowly release the oil, providing prolonged antimicrobial activity in food packaging films [49]. These techniques address the fundamental issue that neat TTO would otherwise just separate out or volatilize quickly from a water-based food or coating. Nanoemulsion and encapsulation systems typically exhibit encapsulation efficiencies ranging from approximately 60% to 95%, depending on formulation composition and processing conditions. Particle size distributions commonly fall within 50–300 nm, with zeta potential values (±20–40 mV) indicating colloidal stability. These parameters directly influence dispersion behavior, antimicrobial contact efficiency, and release kinetics.

The hydrophobic nature of TTO also means it has a high affinity for food components like fats and proteins. In complex food matrices, essential oil molecules can become solubilized in fat droplets or bound to proteins, which can reduce the amount of free TTO available to act on microbial cells [50]. High-fat foods tend to require higher EO concentrations to achieve the same antimicrobial effect observed in leaner systems. The fat can essentially sequester lipophilic compounds like terpene alcohols. Proteins, especially if denatured or in high concentration, may interact with terpenes via hydrophobic binding sites [51]. As a result, when using TTO in, say, meat or a dairy product, one must account for this and possibly use a higher dose or a delivery system that targets the aqueous phase. Otherwise, the oil might largely embed in the fat or protein matrix, lowering the concentration in the water phase where many bacteria reside.

The inherent hydrophobicity of TTO precludes direct aqueous dispersion and represents a fundamental formulation challenge for food safety applications. The mode of incorporation, whether applied as neat oil or in an emulsified or encapsulated form, critically determines the distribution of bioactive terpene constituents within the food matrix and, consequently, governs antimicrobial efficacy. Appropriate formulation strategies employing emulsifiers, biopolymer carriers, or nanostructured delivery systems are therefore essential to overcome solubility limitations and achieve homogeneous distribution of TTO throughout aqueous or semi-aqueous food systems. When successfully engineered, emulsified and encapsulated TTO formulations have demonstrated enhanced antimicrobial potency relative to bulk oil at equivalent or lower concentrations, an effect attributed to increased interfacial contact area and improved accessibility to microbial targets. Conversely, inadequate dispersion results in phase separation, uneven terpene distribution, and localized sensory defects, including concentrated oily off-notes, while simultaneously compromising the antimicrobial coverage that justifies TTO’s use as a natural preservative. Formulation quality is therefore not merely a physicochemical consideration but a determinant of both the safety performance and sensory acceptability of TTO-containing food products.

### 2.4. Volatility and Aroma

Tea tree essential oil is a volatile oil, meaning its constituents readily evaporate at ambient temperatures, a defining trait of essential oils [52]. This volatility is reflected in TTO’s measurable vapor pressure, which is about 2.1 kPa at 25 °C (approximately 15–16 mmHg). While lower than the vapor pressure of water at 25 °C (~3.2 kPa), this is still sufficiently high that TTO will slowly vaporize when exposed to air. In a practical sense, an open container of TTO will lose weight over time as the lighter terpenes evaporate, and any product containing TTO may release its aroma into the headspace.

The volatility of TTO means that it has a strong aroma that can quickly permeate its surroundings. The scent is often described as spicy, camphoraceous, or medicinal, owing to compounds like terpinen-4-ol and α-terpinene. In food applications, this strong odor and flavor potential is a double-edged sword. At low levels, it can contribute to a fresh, eucalyptus-like note that might be acceptable or even desirable in certain products. For instance, herbal teas, chewing gums, or mouthwash-like applications. However, at higher concentrations, TTO’s flavor is quite pungent and bitter, which could spoil the organoleptic qualities of most foods [53]. Thus, volatility governs not only how TTO is delivered as an antimicrobial but also how consumers perceive its presence sensorially. Managing the aroma impact often means using the minimal effective concentration and perhaps pairing TTO with complementary flavors (mint, spice, etc.) if used directly in a food.

One significant advantage of TTO’s volatility is that it can act in the vapor phase to inhibit microorganisms in air or on surfaces. Unlike non-volatile preservatives, TTO does not have to be in direct liquid contact with a microbe to exert some effect; its evaporated molecules can diffuse and reach microbial cells. This is particularly relevant for food packaging and storage. For example, in active packaging, sachets or coating films containing TTO can slowly release vapor that fills the headspace of a package, providing an atmosphere that suppresses mold and bacterial growth on the food surface [54]. Research has demonstrated that TTO vapors can inhibit common food spoilage fungi. TTO vapor significantly reduced *Botrytis cinerea* mold growth on strawberries in storage [55]. Treated strawberries exposed to TTO at around 0.3–0.9 g/L air for a few hours showed delayed onset of gray mold and maintained better sensory quality over several days. Similarly, TTO and other essential oil vapors have been reported to curb fungal decay in fruits and bread. This demonstrates a possible application with fumigation or vapor-phase delivery of TTO in produce storage or bakery packaging to extend shelf life without direct contact.

However, volatility also means TTO can be lost from a food system over time. If TTO is applied to an open food surface (e.g., as a spray or dip), a substantial fraction may evaporate into the environment, diminishing its residual antimicrobial effect and slowing release and volatilization. For instance, coating packaging material with TTO-loaded microcapsules can achieve a sustained slow release of vapor rather than a rapid flash-off of the oil. In a closed package, volatilized TTO is partly retained in the headspace, so it is not entirely lost and contributes to an inhibitory atmosphere. But in an open storage scenario or high-airflow conditions, maintaining an effective concentration of TTO vapor is challenging. Controlled-release behavior in nano-delivery systems typically follows biphasic kinetics, characterized by an initial burst release (20–40% within the first few hours) followed by sustained diffusion over extended periods [56]. This release profile is critical for maintaining antimicrobial concentrations while minimizing rapid volatilization losses.

From a food safety and preservation standpoint, the volatility of TTO is beneficial in applications like: (a) Active packaging, where TTO volatiles provide continuous antimicrobial action and even penetrate small crevices on food surfaces, and (b) Surface sanitation, where TTO vapors can reduce airborne or surface microbial loads in storage environments. Conversely, in liquid foods or high-moisture foods stored in unsealed conditions, volatility means TTO might dissipate before it can significantly act on microbes, or its concentration might drop below effective levels over time.

One must also consider regulatory and safety aspects: the fact that TTO volatiles will be inhaled or contribute to flavor means usage levels need to be controlled to avoid consumer aversion or potential respiratory irritation. Generally, only very small amounts of TTO are needed to achieve an antimicrobial effect in vapor form; often a few µL per liter of headspace can show activity against molds. This is fortunate, as it helps stay below sensory detection thresholds in many cases. Monitoring the peroxide value and compositional changes in TTO in such applications is also important, because prolonged volatilization can enrich certain components in the residue and possibly in the headspace as well (e.g., the more volatile fractions evaporate first, altering the oil’s makeup).

TTO’s volatility, therefore, is a key property that enables vapor-phase interventions for food preservation but also necessitates formulation strategies to control its release. It influences how we package foods containing TTO, often needing sealed packaging to trap the vapors, and how frequently active packaging might need to be replaced or replenished as the oil evaporates. The volatile nature, combined with the oil’s potent aroma, means that achieving the right balance between microbial inhibition and sensory acceptance is critical when leveraging TTO in food systems.

### 2.5. Oxidative Stability

Like many essential oils, tea tree oil is subject to oxidative degradation upon exposure to air, light, and heat. Oxidation is a chemical process where reactive oxygen, from air or other sources, interacts with the oil’s constituents, leading to the formation of new compounds such as peroxides, epoxides, alcohols, or acids. For TTO, which is rich in unsaturated terpenes, oxidation is a particular concern because it can not only diminish the oil’s antimicrobial efficacy but also produce by-products that may be undesirable or even harmful. For example, some oxidation products are strong sensitizers that can cause allergic reactions to skin contact. In a food context, oxidation could potentially lead to off-flavors or reduced preservative function.

The major TTO components vary in their susceptibility to oxidation. Terpinen-4-ol (the primary active) is a tertiary alcohol and relatively stable to mild oxidation that tends to remain constant unless oxidation is severe. In contrast, the terpene hydrocarbons (e.g., α-terpinene, γ-terpinene, terpinolene) are more prone to autoxidation. When exposed to air and light over time, α-terpinene and γ-terpinene gradually oxidize to form compounds like p-cymene (an aromatic terpene) and various peroxides. Indeed, p-cymene often increases in aged or poorly stored TTO as it can be an oxidation product of the terpinene isomers. One study that stored TTO in opened bottles over 12 months, with periodic exposure to air/light simulating consumer use, found little change in terpinen-4-ol content but observed a decline in α- and γ-terpinene and a corresponding rise in p-cymene levels, along with a measurable increase in peroxide value [57]. The peroxide value of fresh high-quality TTO is typically <10 micro equivalents O_2_, a measure of reactive peroxide content, but this can climb as oxidation progresses. Over prolonged or intense oxidation, new oxygenated compounds appear, some of which are known allergens or irritants. For example, ascaridole (a peroxide) and 1,2,4-trihydroxymenthane (a triol) have been detected in heavily oxidized TTO and are implicated in allergic contact dermatitis cases.

Given its oxidation-prone components, TTO should be stored in air-tight, light-resistant containers, in cool conditions to preserve its quality. Amber glass bottles (to block UV light) and filling the headspace with inert gas (or minimizing headspace) are common practices to slow oxidation. Antioxidants like α-tocopherol (vitamin E) or rosemary extract are sometimes added to essential oils to extend shelf-life. In the context of food applications, if TTO is incorporated into a product or packaging, the formulation might include antioxidants to protect not just the food but also the integrity of the oil itself. For example, an edible antimicrobial coating might combine TTO with a natural antioxidant to prevent the oil from oxidizing during the product’s shelf life, thereby maintaining its antimicrobial potency and avoiding the development of off odors from oxidation products.

Stored TTO can remain relatively stable for a considerable period. The 12-month study mentioned above showed no appreciable degradation in a well-stored oil, aside from minor expected changes [58]. However, in less ideal conditions, say a transparent spray bottle regularly opened, TTO could oxidize significantly within months. The rate of oxidation also increases with temperature; hence, high-temperature processing or storage of foods containing TTO might accelerate breakdown. In a food safety scenario, this means the timing of TTO addition is important: adding it at the end of cooking (to avoid thermal degradation) or using encapsulated forms that release after cooling can help. If TTO is used in a packaging film and that film is subjected to heat (e.g., during sealing or if used in hot-fill processes), one must ensure the oil remains effective and does not form harmful compounds. Encapsulation within polymeric or lipid-based nanocarriers has been shown to significantly enhance oxidative stability, reducing peroxide formation rates and extending functional shelf life by up to 2–3-fold compared to free oil systems [59].

Oxidation can alter the antimicrobial activity of TTO in complex ways. Moderate oxidation might not severely impact the antimicrobial power if terpinen-4-ol and other key actives remain high. In fact, some oxidation products (like peroxides) have antimicrobial properties of their own, although they tend to be less studied and could be more toxic or unstable. However, extensive oxidation that reduces the content of monoterpene alcohols and increases inert or less-active compounds will likely diminish efficacy. Moreover, oxidized oil often has a harsher smell, which could be problematic sensorially. In topical medicinal use, oxidized TTO is avoided because of allergy risks, but in foods, the bigger concern would be rancid or off flavors.

For applications in food packaging, one needs to consider that an oxidized oil may not provide the same level of antimicrobial protection. A study of antimicrobial packaging with essential oils found that the activity dropped if the active oil had oxidized significantly during storage of the package [60]. Thus, researchers sometimes incorporate UV blockers or antioxidants into active packaging films with TTO to keep it fresh. Encapsulation in a polymer matrix can also inherently slow oxidation by limiting oxygen exposure.

Regulatory bodies typically expect that if an essential oil is used in a food contact material or directly in a food, it should not undergo chemical changes that produce unsafe substances [61]. Therefore, demonstrating the oxidative stability of TTO under the intended use conditions is important. If oxidation products form, a safety assessment might be needed to ensure they are not harmful if ingested. Moreover, regulatory frameworks do not consistently define exposure limits for tea tree oil across different application modes (e.g., direct food additive versus migration from packaging), creating ambiguity in compliance and safety evaluation. So far, the known major oxidation products of TTO, such as ascaridole, are present in very low amounts in moderately aged oils and are more of a concern for skin exposure than ingestion at the trace levels likely in foods. Under appropriate storage conditions, tea tree essential oil can remain chemically stable for at least 6 months and, in some controlled storage studies, for up to 12 months without appreciable oxidative deterioration, as indicated by limited changes in p-cymene content and peroxide value. However, exposure to air, light, and heat markedly accelerates oxidation; under poorly controlled conditions, substantial peroxide formation and compositional shifts in oils may occur within days [62]. From a practical perspective, this stability window is generally sufficient to cover the shelf life of most refrigerated fresh foods, including fresh poultry (1–2 days), fresh meat cuts (3–5 days), pasteurized milk (approximately 5–7 days after opening), and many leafy vegetables (about 7–10 days). Nevertheless, the relevant benchmark in food applications is not only the stability of neat TTO during storage, but also its retention after incorporation into coatings, emulsions, or packaging systems, where volatilization and matrix interactions may shorten functional stability [63].

In summary, TTO is moderately stable when protected but will oxidize over time with exposure. The extent of oxidation can influence safety and sensory properties. For successful use in food preservation, strategies to maintain TTO’s stability, such as proper storage, formulation with stabilizers, and using protective delivery systems, are employed so that the oil retains its antimicrobial potency throughout the product’s shelf life. Additionally, monitoring indicators of oxidation (like peroxide value or changes in aroma profile) can be part of quality control when TTO is used in food processing or packaging, ensuring that consumers get the intended benefit (microbial inhibition) without negative side effects (off-taste or allergens).

### 2.6. Interactions with Food Matrices and the Needs for Nano-Enabled Delivery

The real-world efficacy of tea tree oil in a food system is not determined by the oil’s intrinsic antimicrobial activity alone, but also by how it interacts with the components of that food matrix. Foods are complex and can contain fats, proteins, carbohydrates, water, and various colloidal structures, all of which can influence the distribution and activity of added antimicrobials like TTO. Several factors in foods can diminish the apparent activity of TTO compared to a simple laboratory broth test. Understanding these interactions is critical for designing effective applications of TTO in food safety.

TTO components are lipophilic and will preferentially dissolve into lipid phases [64]. In high-fat food (e.g., cheese, sausage, bakery products with fat, or oil-in-water emulsions like mayonnaise), a large portion of the TTO added may partition into the fat portion. This has two implications: (1) it can protect certain bacteria that reside in the aqueous phase or at water–fat interfaces because the active compounds are drawn away into the bulk fat phase, and (2) the fat can act as a solvent for TTO, possibly reducing direct contact between the oil and microorganisms. Studies have shown that higher-fat foods often require larger doses of essential oils to achieve the same antimicrobial effect as low-fat foods [65]. For example, an essential oil that effectively inhibits *Listeria* in a lean fish broth might need a much higher concentration in fatty minced meat, as much of the oil disappears into the fat of the meat. In meat and dairy, fat droplets also physically encapsulate bacteria or create protective niches, while fat coating on microbial cells can impede the contact or uptake of antimicrobial agents. In one scenario, the fat in a reformulated low-fat sausage was observed to have less protective effect on microbes, resulting in stronger antimicrobial activity of additives, whereas higher-fat sausage required more additives to see an effect. Thus, when using TTO in a fatty food, one might need to increase concentration or use formulation tricks like pre-mixing the oil with an emulsifier so that it does not immediately vanish into the fat.

Proteins in foods (such as milk proteins, egg proteins, or gluten in dough) can bind flavor and aroma compounds, including terpenes. Phenolic compounds and terpenoids often have an affinity for protein, potentially through hydrophobic interactions or even covalent binding (if the oil contains any reactive aldehydes, though TTO is mostly terpenes and alcohols). While terpinen-4-ol and others are not strongly phenolic, they are still hydrophobic enough to stick to proteins. This binding can reduce the free concentration of TTO components that are able to interact with microbial membranes. For example, if TTO is added to a protein-rich beverage (like a protein shake or soup), some of its molecules might get absorbed onto the protein surfaces, thus less is available in solution to act on bacteria. Carbohydrates generally have less of an affinity unless they form inclusion complexes (cyclodextrins can encapsulate small terpenes, intentionally used in some cases). But in solid foods, carbohydrates (starches, fibers) might just physically occlude oils or change the microstructure in a way that the oil is trapped in certain phases. While higher concentrations are often required to overcome matrix-related losses, the safety implications of such elevated doses remain insufficiently defined, particularly in relation to acceptable daily intake and sensory tolerance limits.

Water activity (a_w_) and pH of the food also interact with how well an essential oil works. TTO’s activity might improve at lower a_w_ or lower pH in some cases, as stressed bacteria are more susceptible [66]. But if the food is very dry, the distribution of the oil could be uneven, and some of it may volatilize more readily. If pH is very low (like in a pickle or a fermented dairy), TTO does not ionize (it is mostly non-polar), so pH does not directly affect the oil, but the overall antimicrobial hurdle is changed—at low pH, maybe less oil is needed as bacteria are already weakened. This can be leveraged by combining TTO with other hurdles (like mild acidity or mild heat) to get a synergistic kill effect.

The structure of the food, including emulsion, gel, and solid matrix, matters as well. In emulsified foods (dressings, sausages), as noted, partitioning between phases is key. In solid foods or biofilms on food surfaces, the oil has to diffuse to reach microbes. TTO applied on a fruit surface might not penetrate deeply if the fruit has waxy cuticles [67]. Conversely, in a porous food like bread, TTO vapors might travel through pores and reduce mold internally. Some researchers have studied TTO in edible coatings on produce, with the coating matrix (often polysaccharide or protein-based) controlling the release of TTO onto the fruit surface over time [68]. A too-tight matrix might retain the oil too much, whereas a very open matrix might let it evaporate too quickly. Thus, tailoring the delivery matrix (e.g., a glycerol-plasticized alginate film vs. a zein protein coating) can influence how effectively TTO migrates to where microbes are. Structure–activity relationships indicate that smaller particle sizes enhance antimicrobial efficacy by increasing surface area and facilitating closer interaction with microbial membranes. Similarly, surface charge plays a critical role; positively charged nanocarriers exhibit stronger adhesion to negatively charged bacterial cell walls, improving antimicrobial performance. These relationships highlight that functional outcomes are not solely concentration-dependent but are strongly influenced by nanocarrier physicochemical properties.

A recurring finding is that the antimicrobial efficacy of essential oils, including TTO, is often reduced in real food systems compared to laboratory broth. Burt (2004) noted that this is because the factors (fat, protein, salt, etc.) in foods can all interfere or require the oil to be used at higher concentrations [69]. This gap means that when formulating a food preservative system, one cannot rely solely on minimum inhibitory concentration (MIC) values determined in nutrient broth; one must test in the actual food matrix. For instance, if TTO at 0.02% *v*/*v* prevents growth of *E. coli* in laboratory media, it might need 0.1% or more to do the same in a salad dressing with oil and vinegar, or it might be ineffective until 0.5% in a rich stew [70]. High concentrations, however, risk making the food taste medicinal. Therefore, a practical approach is often to use TTO in combination with other preservative hurdles (mild heat, acidity, or other natural antimicrobials) so that each can be at a lower concentration. Some studies have shown synergistic effects, such as TTO working better when used along with a mild thermal treatment on fruit, as heat may make cell membranes more permeable to the oil.

To overcome matrix interactions, encapsulation techniques are again useful. By encapsulating TTO in a carrier (like lipid nanoparticles or polymer fibers), one can sometimes target the release of the oil to certain phases or delay its release until after a processing step. For example, a pH-responsive encapsulation could hypothetically keep TTO bound during high-fat cheese ripening (neutral pH) but release it when the product is consumed or when pH drops slightly due to microbial action, thereby sparing it from binding to fat early on [71]. There is ongoing research into such smart delivery systems for essential oils in foods.

In conclusion, the interactions of TTO with food matrices mean that formulators must consider the food’s composition when determining the usage level and method of incorporation. High-fat and high-protein foods pose the greatest challenges, often requiring higher doses or innovative delivery methods (such as nanoemulsions) to achieve the desired antimicrobial effect. The goal is to maximize the availability of TTO’s active components at the sites where microbes reside (often the aqueous phase or surface of foods) while minimizing losses to the food matrix. Successful case studies include using emulsified TTO in low-fat soups and vapor-phase TTO in bread packaging. Collectively, these matrix interactions explain why nano-enabled delivery systems have become increasingly attractive. By protecting TTO within nanoscale carriers, premature binding to proteins and lipids can be reduced while maintaining higher concentrations of bioactive compounds at microbial target sites.

### 2.7. Extraction, Purification, and Analytical Methods for Characterization and Quality Assurance

**Figure 3:**
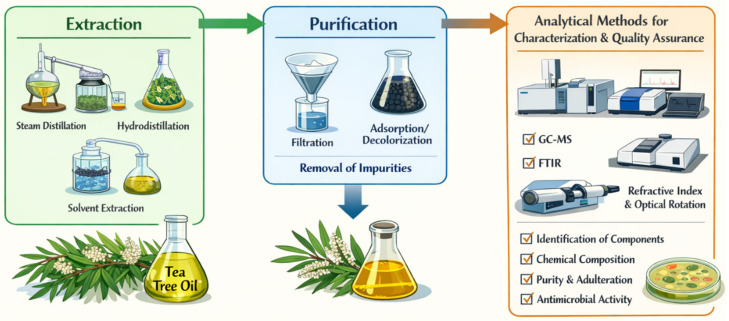
Processing and characterization strategies for TTO (created by BioRender and modified by Figure Labs).

The quality, composition, and functional performance of tea tree essential oil (TTO) are strongly influenced by extraction, purification, and analytical characterization processes, which collectively determine its suitability for food applications [72]. Variations in processing conditions can significantly alter terpene composition, particularly the concentration of terpinen-4-ol, thereby affecting antimicrobial efficacy and compliance with international standards such as ISO 4730 [7,26]. Therefore, integrating production and quality assessment within a unified framework is essential for ensuring consistency, safety, and regulatory acceptance. Figure 3 shows the integrated workflow illustrating extraction, purification, and analytical characterization strategies for tea tree oil (*Melaleuca alternifolia*). Extraction methods include steam distillation, hydrodistillation, and solvent extraction, yielding crude essential oil rich in terpene constituents. Post-extraction purification via filtration and adsorption enhances oil clarity and removes non-volatile impurities. Comprehensive quality assurance is achieved through GC–MS and FTIR analyses, complemented by physicochemical parameters. These analytical approaches enable precise compositional profiling, authenticity verification, impurity detection, and functional assessment, including antimicrobial activity.

TTO is predominantly obtained from *Melaleuca alternifolia* leaves via steam distillation, which remains the industrial standard due to its scalability and ability to preserve the natural terpene profile [7]. In this process, steam volatilizes essential oil components, which are subsequently condensed and separated based on immiscibility with water. Despite its widespread use, steam distillation may lead to partial degradation or loss of thermolabile and highly volatile compounds under prolonged heating [73]. Alternative techniques, such as hydro-distillation, are less favored for high-quality production due to potential hydrolytic reactions and lower efficiency [74]. In contrast, advanced approaches such as supercritical CO_2_ (SC-CO_2_) extraction offer improved selectivity and operate under moderate temperatures, enabling the recovery of high-purity oils with enhanced retention of bioactive constituents and minimal solvent contamination [75]. Emerging green technologies, including ultrasound- and microwave-assisted extraction, have also demonstrated increased efficiency and reduced processing time, although their industrial scalability remains limited [76].

Following extraction, purification, and fractionation processes are often employed to optimize TTO composition and meet quality standards. Molecular distillation is widely used to refine essential oils by separating components based on volatility differences under reduced pressure, allowing enrichment of terpinen-4-ol and reduction in undesirable compounds such as 1,8-cineole [77]. At the laboratory scale, column chromatography enables detailed fractionation and isolation of individual terpenes for mechanistic or formulation studies, although its application in industrial processing is constrained by cost and complexity. These purification strategies are critical for improving antimicrobial consistency, sensory acceptability, and regulatory compliance in food-related applications.

A comprehensive analysis of TTO’s physicochemical properties and composition is essential, especially when TTO is used in food-related applications that demand consistent quality and regulatory compliance. The primary analytical approaches for TTO characterization are chromatographic methods (especially GC-MS) for chemical profiling, and spectroscopic methods (like FTIR) for rapid identification and adulteration detection. Additionally, physical assays (density, refractive index, and optical rotation as mentioned) are routinely used for quality control.

Gas Chromatography–Mass Spectrometry (GC-MS) is considered the gold standard for essential oil analysis, and virtually all detailed TTO composition data in the literature come from GC-MS [78]. In this technique, TTO (neat or in solution) is injected into a gas chromatograph; the volatile constituents are separated on a capillary column and then identified by their mass spectra and retention times (often compared against known standards or libraries). GC-MS can quantify the relative percentages of dozens of components in TTO. For instance, it can confirm that terpinen-4-ol is, say, 40% and 1,8-cineole is 4% in each batch, matching the ISO 4730 profile [47]. This is crucial for ensuring that the TTO used in food or packaging meets the expected specification for efficacy and safety [79]. A GC-MS analysis finds an atypical component or an out-of-range value (e.g., 1,8-cineole at 20%), indicating adulteration or an off-spec source [80]. Food regulatory agencies would also rely on such analyses if TTO were being evaluated as a food additive or contact substance to determine exactly which compounds are present.

High-resolution GC methods (like GC-FID for quantification, GC-MS for identification) are used by the industry for batch certification. Additionally, enantioselective GC can be used to examine the enantiomeric ratios of chiral components (like terpinen-4-ol, which has enantiomers) [81]. This is an advanced test that can differentiate natural TTO from synthetic mixtures. *Melaleuca*-derived terpinen-4-ol has a specific enantiomeric excess, which a racemic composition might suggest synthetic adulteration. Such chiral analysis is a powerful tool because it is nearly impossible for adulterators to mimic the exact chiral signature of natural TTO [82]. In quality control, a combination of GC-MS and enantiomeric GC is recommended for high assurance, especially if TTO is used in medicinal or high-value foods.

For research applications demanding higher resolving power, two-dimensional gas chromatography (GC × GC) has been employed to separate co-eluting components that remain unresolved under conventional one-dimensional GC conditions, yielding a more comprehensive chemical fingerprint of the oil [83]. This approach enables the characterization of minor and trace constituents that may serve as markers of geographical provenance or thermal and oxidative storage history. High-performance liquid chromatography (HPLC) is not routinely applied to TTO analysis, as the oil’s predominantly volatile, non-polar terpene composition falls outside the optimal analyte range of liquid chromatographic methods; however, derivatization-coupled HPLC may be warranted for the targeted analysis of polar degradation products generated during oxidative aging [84]. For quality control and compositional profiling in both research and regulatory contexts, GC-MS remains the standard and sufficient analytical platform.

FTIR spectroscopy, especially in Attenuated Total Reflectance mode, is a rapid, simple method to get a fingerprint of TTO. Each essential oil has a characteristic infrared spectrum based on the functional groups present. TTO’s IR spectrum, for example, shows bands for O–H stretching (from terpinen-4-ol’s alcohol group), C–H stretching of methyl/methylene, and C=C stretching of terpenes, etc [85]. While FTIR lacks the resolution to identify each component, it is very useful for verification and adulteration screening. A pure TTO sample will produce an IR spectrum that can be matched against a reference spectrum. If the sample has been diluted with a vegetable oil (which is a common adulterant tactic to extend essential oils), the IR spectrum will show features of fatty acids (strong carbonyl band around 1740 cm^−1^, etc.) which are absent in genuine TTO [86]. Indeed, studies have shown that FTIR coupled with chemometric models can detect TTO adulteration with cheap carrier oils like soybean or corn oil with high accuracy. For instance, one report noted that a Random Forest–SVM model on FTIR data achieved ~93% accuracy in identifying TTO samples adulterated with corn or soybean oil [87]. This is extremely valuable for quality control because FTIR is quick (a matter of seconds per sample) and does not require solvents or complex preparation. Manufacturers can use FTIR as a screening tool for incoming TTO lots; if an unusual spectrum is observed, they can then do targeted GC-MS to investigate further. FTIR can also monitor changes in TTO due to oxidation. As oxidation progresses, new peaks (e.g., for peroxide O–O or hydroxyl groups from oxidation products) might appear, and baseline shifts might occur [88]. Thus, an FTIR scan might be able to indicate if an oil is significantly aged or degraded (though GC-MS is more definitive in quantifying specific oxidation products). Some researchers have used IR to quantify the extent of adulteration or degradation by building calibration models correlating IR spectral features with known adulterant percentages.

In addition, Raman spectroscopy and Near-Infrared (NIR) spectroscopy have been explored for essential oils. NIR can even be used through packaging to verify if oil inside a closed container is authentic. For example, a study using NIR could detect tea tree oil adulteration by scanning the bottle without opening it, a convenient method for ensuring the integrity of packaged oils [89].

Another advanced approach mentioned in the literature is Nuclear Magnetic Resonance (NMR) spectroscopy. While NMR is not routine for every batch due to cost, it has unique strengths. A recent development introduced a C-NMR method to detect vegetable oil adulterants in essential oils [90]. This method could unambiguously spot even subtle adulteration without needing chemometric analysis, because the carbon backbone signals of terpenes are distinct from those of triglycerides. For TTO, an NMR profile could also be used to quantify major components in an absolute sense (whereas GC is often area-percent). If TTO were to be used as a food ingredient with a need for precise labeling, one might use quantitative NMR (qNMR) to determine absolute terpinen-4-ol content in mg/mL, for instance.

Measurement of density, refractive index, and optical rotation constitutes a classical and cost-effective approach to the rapid assessment of essential oil identity and purity. These physicochemical parameters are particularly valuable for detecting gross adulteration: the addition of extraneous fatty oils, for instance, produces characteristic deviations in both density and refractive index that fall outside established reference ranges. Compliance with pharmacopeial specifications for these properties is generally required for food-grade essential oil certification. Oxidative stability is most directly assessed through the peroxide value (PV), a parameter adapted from edible fat and oil analysis that quantifies reactive oxygen species via titrimetric determination. A low PV in freshly processed TTO, typically below 10 µeq O_2_/kg, confirms minimal oxidative degradation and is indicative of adequate storage and handling conditions [91]. Elevated PV values, conversely, signal the accumulation of primary oxidation products and warrant concern regarding both the antimicrobial performance and the safety profile of the oil, given the established sensitizing and irritant potential of terpene-derived peroxides and secondary oxidation metabolites.

For nano-delivery systems, additional characterization techniques are essential, including dynamic light scattering (DLS) for particle size distribution, zeta potential analysis for colloidal stability, and electron microscopy (TEM/SEM) for structural morphology. Encapsulation efficiency is typically quantified using spectroscopic or chromatographic methods by comparing total and free oil fractions [92]. When TTO is incorporated into food or packaging, analytical methods are used both in formulation and in end-product testing. Gas chromatography can be used to measure how much of the TTO (and which components) remain in a food product over time (for example, to comply with regulations or to understand release kinetics). If TTO is applied in packaging film, headspace GC-MS can analyze the package atmosphere to quantify volatile release. Likewise, migration tests might be done to ensure that TTO components do not migrate through packaging at levels beyond legal limits if used in food contact materials. Regulatory agencies might require such data. For instance, the EU treated TTO components as flavorings or active packaging substances and set specific migration limits [93].

**Figure 4:**
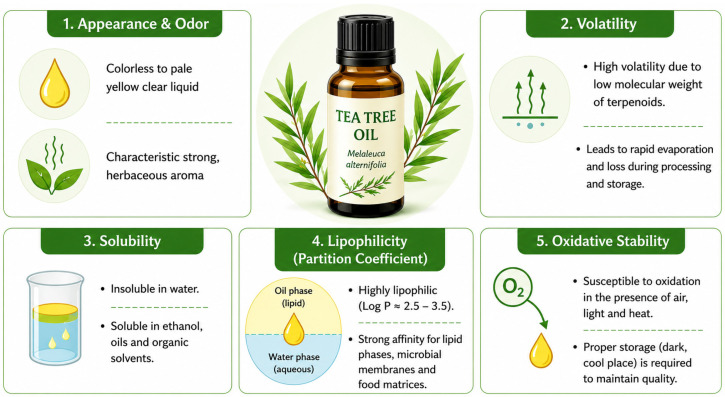
Physicochemical properties of TTO (created by BioRender and modified by Figure Labs).

In summary, robust analytical characterization of tea tree oil is indispensable to its application in food safety. GC-MS provides the detailed composition, ensuring the oil used is genuine and of the right chemotype (crucial for efficacy). FTIR and related spectroscopic methods offer rapid screening for authenticity and quality (important for routine QA/QC). Physical and chemical assays, including measurements of refractive index (RI), density, peroxide value (PV), and related parameters, provide additional confidence that the oil will perform and remain stable under expected conditions during processing, storage, and application (Figure 4).

By employing these analytical tools, producers can guarantee that the TTO in their antimicrobial formulation is of high quality, meaning it contains the intended active compounds in proper amounts, has not been adulterated or degraded, and will thus reliably contribute to food preservation as designed. These processing and analytical strategies are particularly important in nanotechnology-enabled delivery systems, where slight variations in TTO composition can significantly influence encapsulation efficiency, release behavior, and antimicrobial performance. This analytical vigilance ultimately supports both the efficacy of TTO in real-world applications and the safety of the final food products for consumers.

## 3. Antimicrobial and Antibiofilm Activities of Tea Tree Oils (TTO)

**Figure 5:**
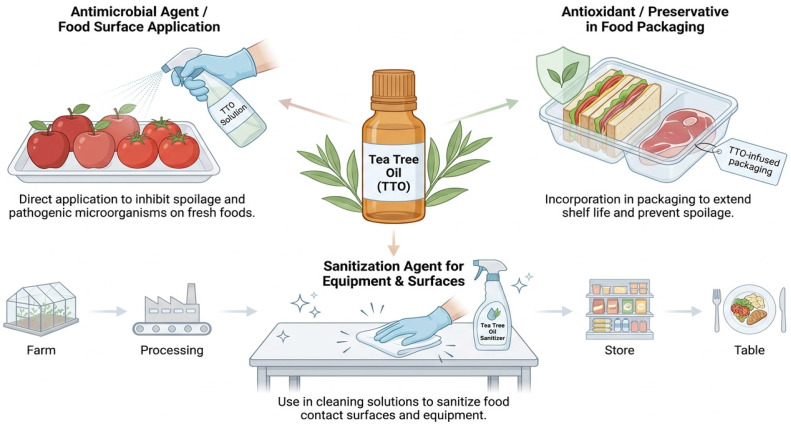
TTO applications in the food safety system (created by BioRender and modified by Figure Labs).

Tea tree essential oil (TTO) exhibits broad-spectrum antimicrobial activity against a variety of bacteria, fungi, and even certain viruses. These antimicrobial effects, coupled with anti-quorum-sensing and antibiofilm properties, make TTO a compelling natural agent for food safety applications (Figure 5).

This section reviews TTO’s activity against Gram-positive and Gram-negative bacteria, its efficacy on yeasts and molds, and its antiviral potential, alongside the underlying mechanisms of action (membrane disruption, oxidative stress induction, enzyme inhibition, quorum sensing interference, etc.). Minimum inhibitory concentrations (MICs), time-kill studies, and comparisons with other essential oils, with a particular focus on foodborne pathogens and biofilm-related food safety concerns, are emphasized.

### 3.1. Antibacterial Activity Against Gram-Positive and Gram-Negative Bacteria

TTO is broadly active against many bacteria, typically exhibiting inhibitory effects at concentrations ≤ 1% *v*/*v* for most species (Table 5).

Although antimicrobial efficacy is typically observed within the range of 0.05–1% (*v*/*v*), these concentrations may exceed acceptable sensory or toxicological thresholds in food systems. The absence of integrated dose–response and safety data limits the translation of in vitro antimicrobial performance into practical food applications. For example, *Staphylococcus aureus* (a Gram-positive pathogen) is usually inhibited by TTO at ~0.25–0.5% *v*/*v*, and even methicillin-resistant *S. aureus* (MRSA) strains show similar susceptibility ranges (MIC on the order of 0.25–0.312% *v*/*v*). Carson et al. (2006) reported that while most bacteria are susceptible to ≤1.0% TTO, some organisms require higher concentrations; notably, commensal skin *Staphylococci*, *Enterococcus faecalis*, and *Pseudomonas aeruginosa* have MICs > 2% [7]. Indeed, *P. aeruginosa*, a Gram-negative bacterium known for its robust outer membrane and efflux pumps, can exhibit MIC values as high as 8% *v*/*v* in some cases. By contrast, other Gram-negatives like *Escherichia coli* and *Salmonella enterica* are inhibited at lower levels (e.g., MIC ~0.08–0.5% in many strains), although some resilient strains can require up to 2%. Systematic surveys of TTO activity against clinically and food-relevant pathogens have established MIC values for *Escherichia coli* in the range of 3.1–3.4 mg/mL (0.35–0.40% *v*/*v*) and for *Salmonella* Typhimurium at approximately 6.2 mg/mL (0.70% *v*/*v*), confirming meaningful bacteriostatic and bactericidal activity while highlighting that effective concentrations are generally higher than those required for phenolic-rich essential oils such as oregano [10]. This differential susceptibility broadly reflects differences in cell envelope architecture: Gram-positive organisms, which lack an outer membrane barrier, tend to exhibit greater sensitivity to TTO’s terpene constituents than Gram-negative species, although notable exceptions exist. *Pseudomonas aeruginosa*, for instance, displayed pronounced intrinsic resistance attributable to the low permeability of its outer membrane, and effective activity against this organism typically requires the co-application of membrane-permeabilizing agents. The bacteriostatic versus bactericidal nature of TTO is concentration-dependent: at doses at or above the MBC, rapid cell death predominates, whereas sub-inhibitory concentrations elicit a bacteriostatic response characterized by growth retardation without complete sterilization, a distinction with practical implications for dose selection in food preservation contexts.

Nanostructured delivery significantly modulates these intrinsic activity parameters. Nanoemulsified TTO formulations have consistently demonstrated 2–5-fold reductions in MIC values relative to equivalent concentrations of free oil, an enhancement attributed to improved aqueous dispersion, increased bioavailability at the microbial interface, and the sustained-release kinetics that prolong effective terpene exposure [9]. These findings underscore the formulation dependency of TTO antimicrobial performance and reinforce the translational relevance of engineered delivery systems for food safety applications.

TTO has demonstrated efficacy against major Gram-positive food pathogens such as *Listeria monocytogenes* and *S. aureus*. In an in vitro study, *L. monocytogenes* showed excellent susceptibility to TTO, with low MIC values and large inhibition zones reported. Shi et al. (2018) [96] found that TTO effectively inhibited *L. monocytogenes* in culture and even in a food model (fresh cucumber juice), where a time-kill assay confirmed complete growth inhibition at refrigerated (4 °C) and ambient (25 °C) conditions. *S. aureus* (including toxin-producing food isolates) is similarly susceptible: typical MICs are around 0.5% or below, and TTO can kill even antibiotic-resistant *S. aureus* [96]. For instance, Thompson et al. (2008) reported that TTO had potent anti-staphylococcal activity against both methicillin-sensitive and methicillin-resistant *S. aureus*, with no significant differences in MIC [97]. This is encouraging for food safety, as *S. aureus*, a cause of food intoxications via heat-stable enterotoxins, could potentially be controlled by TTO where conventional antibiotics fail. Additionally, TTO shows activity against *Bacillus cereus* (MIC on the order of 0.3%), which is another spore-forming food pathogen, though its spores themselves (and those of other spore-formers) are likely much more resistant (spores generally require harsher treatments to inactivate) [98].

Gram-negative food pathogens, including *Salmonella* spp., pathogenic *E. coli* (e.g., O157:H7), *Campylobacter jejuni*, and others, are a key target for natural antimicrobials. TTO has shown inhibitory effects on many such organisms, though often requiring moderately higher concentrations relative to Gram-positives [99]. In one comparative study screening 21 essential oils against 10 strains each of *Salmonella enterica* and *Listeria monocytogenes*, tea tree oil was identified among the top five most effective oils alongside oregano, cinnamon, clove, and thyme in inhibiting these pathogens [100]. TTO’s MIC values in that study ranged up to the higher end, reportedly ~20 µL/mL for some strains, which was higher than oregano’s ~0.6 µL/mL, indicating that while TTO is active, phenolic oils like oregano and clove can achieve the same effect at lower doses. This aligns with the known strong antimicrobial potency of phenolics (e.g., carvacrol, eugenol) compared to terpene alcohols like terpinen-4-ol. Notably, the same study noted that the relatively high MICs of TTO might limit its practical use in foods, as achieving effective concentrations could adversely affect organoleptic properties [101]. Nonetheless, TTO has strengths as it appears especially effective against certain Gram-negative strains. For example, cinnamon and clove oils are very potent against *Listeria*, whereas tea tree oil was observed to be more effective against *Salmonella* in some head-to-head tests. Puvača et al. (2021) also found that TTO exhibited the strongest overall antimicrobial activity among several commercial oils tested, showing MICs of ~3.1 mg/mL for *E. coli* and ~2.7 mg/mL for a reference *E. coli* ATCC 25922 [10]. In practical terms, incorporation of TTO into food systems has yielded promising results: for instance, adding TTO to fresh produce juices or edible coatings significantly inhibits *E. coli* and *Salmonella* growth. These findings suggest that TTO could serve as a natural preservative to reduce Gram-negative foodborne pathogens. However, optimization (or combination with other hurdles) may be needed to overcome the protective outer membrane of Gram-negatives.

The antimicrobial action of TTO is typically rapid. Time–kill assays demonstrate that increasing TTO concentrations accelerate the killing of bacteria and prolong the lag phase of any survivors. For example, *S. aureus* exposed to TTO at its MIC or 2 × MIC suffers a dramatic viability loss within minutes to hours. Similarly, *E. coli* treated with ~0.25% TTO showed significant population reductions, though complete killing might require a slightly higher concentration or longer exposure (owing to *E. coli*’s slightly higher MIC in that case). When TTO was incorporated at 0.2–0.3% in a food matrix (cucumber juice), it successfully prevented the growth of both *L. monocytogenes* and *E. coli* over several days at both 4 °C and 25 °C, indicating bacteriostatic/bactericidal preservation in real food conditions [96]. Dong et al. (2019) note that gels containing ~5% TTO have been shown in some clinical studies to reduce inflammatory acne lesions (papules, pustules), comedones, and overall lesion count [102].

Compared to other essential oils, TTO’s bactericidal efficacy is often high, though not always the absolute highest. Oregano oil, rich in carvacrol, and thyme oil, rich in thymol, sometimes outperform TTO in MIC and kill rate against certain bacteria. However, TTO still ranks among the most effective oils in numerous studies, and it has the advantage of a distinct chemical profile (dominated by terpinen-4-ol rather than phenolics), which can make it a useful alternative or complementary agent to the more pungent phenolic oils. TTO’s components may synergize with other antimicrobials; for instance, combinations of TTO with conventional antibiotics or with other oils have shown additive effects against antibiotic-resistant bacteria. Overall, the evidence indicates that TTO is a potent antibacterial essential oil, capable of inhibiting a wide range of food-related bacteria, though required concentrations can vary by species and strain.

### 3.2. Antifungal Activity

In addition to bacteria, tea tree oil exhibits significant antifungal properties against yeasts (e.g., *Candida* spp., *Saccharomyces*) and filamentous molds (*Aspergillus*, *Penicillium*, dermatophytes, etc.). Early reports on TTO’s antifungal spectrum were somewhat fragmentary, often limited to *Candida albicans* as a model organism [7]. More comprehensive investigations have demonstrated that a broad range of yeasts and filamentous fungi are susceptible to TTO [53]. Generally, MIC values for most fungi fall between ~0.03% and 0.5% *v*/*v*, and minimum fungicidal concentrations (MFCs) range from ~0.12% to 2%. For example, *Candida albicans* often shows the MIC of TTO on the order of 0.06–0.5% and MFC around 0.25–1% in vitro. Other *Candida* species (e.g., *C. glabrata*, *C. parapsilosis*, *C. tropicalis*) have similar MIC ranges (tens of µg/mL), though some isolates, particularly drug-resistant ones, can require higher concentrations (up to 8% in rare cases) [77]. *Cryptococcus neoformans*, an encapsulated yeast, appears highly susceptible (MIC ≤ 0.1%), whereas *Saccharomyces cerevisiae* (a fermentative yeast relevant to food spoilage) can be more tolerant, with reported MICs up to ~1%. Importantly, many *Candida* clinical isolates, including fluconazole-resistant strains, remain sensitive to TTO, making it a candidate for treating fungal infections or contamination. Wróblewska et al. (2021) determined that the addition of TTO increased the solubility of ketoconazole, improved its release and permeation rate from the vehicles through the synthetic membranes, and enhanced antifungal activity against the tested *Candida* strains (especially in the case of *C. parapsilosis*) [103]. Studies reviewed by Bugarcic et al. (2025) specifically examined drug-resistant *C. albicans*, and ten studies looked at non-*albicans Candida* spp. (e.g., *C. krusei*, *C. kefyr*, *C. lusitaniae*), consistently finding fungistatic/fungicidal effects of TTO albeit at varying concentrations [104].

Tea tree oil (TTO) exhibits antifungal activity against filamentous fungi (molds), although its efficacy varies by species and developmental stage. *Aspergillus niger* has often been reported as relatively resistant, with early studies indicating high minimum fungicidal concentrations (MFCs) of up to 8% (*v*/*v*) required to inactivate spores [105]. This reduced susceptibility is largely attributed to the inherent resistance of dormant conidia, which are known to withstand chemical treatments. However, once germination begins, *A. niger* conidia become significantly more susceptible to TTO, and vapor-phase applications have been shown to inhibit both mycelial growth and sporulation. In contrast, *Aspergillus flavus*, a major foodborne mold associated with aflatoxin production, demonstrates much higher sensitivity, with reported minimum inhibitory concentration (MIC) and MFC values of 1.56 µL/mL (~0.156% *v*/*v*) and 3.12 µL/mL, respectively, indicating strong fungicidal efficacy of TTO. Similarly, *Aspergillus fumigatus* (an opportunistic pathogen and spoilage mold) and *Aspergillus flavus* typically show MICs in the range 0.06–0.5% and MFCs around 1–4%, though again, spore form and hyphal form must be considered. Dermatophyte fungi, such as *Trichophyton* spp., are generally quite susceptible to TTO, with Hammer et al. (2002) [106] noting MICs ~0.03–0.12% for *Trichophyton mentagrophytes* and *T. tonsurans*. TTO is well-known as a topical antifungal (e.g., for athlete’s foot, which is caused by dermatophytes), so its strong activity in that realm is unsurprising. In the context of foods, however, the most relevant molds are those causing spoilage or mycotoxin production (*Penicillium*, *Aspergillus*, *Fusarium*, etc.). Studies have shown TTO can inhibit the growth of *Penicillium* spp. and *Fusarium* spp. as well [107]. For instance, *Fusarium solani* had a 50% respiratory inhibition at a TTO concentration around 0.2–0.25%, hinting at TTO’s fungistatic effect on that plant pathogen.

The antifungal mechanism of TTO appears to parallel its antibacterial action in many respects. Key components like terpinen-4-ol likely disrupt fungal cell membranes and cell walls, leading to leakage of vital intracellular constituents and impairment of vital processes. Carson et al. (2006) reported that TTO inhibited *C. albicans* respiration by ~95% at 1.0% and ~40% at 0.25%, demonstrating a dose-dependent disruption of fungal mitochondrial function [7]. This respiratory inhibition could cause an increase in reactive oxygen species or energy depletion in fungal cells. Electron microscopy studies on TTO-treated *Candida* have shown structural damage to cell membranes, and leakage assays indicate loss of 260 nm-absorbing nucleic acids from yeast cells [106]. Notably, TTO’s main active terpene alcohols may also interfere with the synthesis of ergosterol or other cell wall/membrane components, though this is an area for further research. Some evidence suggests TTO vapors can reduce mold sporulation and spore viability, which is significant for controlling airborne mold spread on stored foods. Practical studies have demonstrated TTO’s antifungal efficacy in situ; for example, treating *Aspergillus*-contaminated substrates with TTO or its nano-formulations significantly reduced mold growth and mycotoxin levels [81]. Additionally, *Candida* biofilms, which are common on food processing surfaces, causing spoilage, or in medical contexts, causing infection, are susceptible to TTO, albeit often requiring higher concentrations than planktonic yeast.

Overall, the fungicidal/fungistatic profile of TTO suggests it could be useful in preventing fungal spoilage of foods (e.g., bakery products, fruits, cheeses) and in inhibiting yeast-mediated spoilage (e.g., in beverages). Table 6 summarizes representative applications of TTO in foods, including the microbial problems addressed, methods of application, formulation strategies, and outcomes in terms of microbial control, shelf-life, sensory acceptance, and feasibility.

Nano-enabled formulations have also demonstrated enhanced antifungal activity against important food spoilage fungi such as *Botrytis cinerea*, *Penicillium expansum*, *Aspergillus niger*, and *Aspergillus flavus*. Improved retention of volatile terpenes and prolonged release enable longer protection during storage, making nanoformulations particularly attractive for active packaging and edible coating applications. Indeed, Tighe et al. (2013) concluded that tea tree oil was the most effective essential oil against a range of foodborne fungal isolates in their tests and identified terpinen-4-ol as the active fraction responsible for strong anti-mold effects [115]. This highlights that TTO not only kills fungi but can also inhibit fungal enzymes. In the same study, terpinen-4-ol showed 80–90% inhibition of key spoilage enzymes produced by foodborne fungi and bacteria [95]. Such enzyme inhibition further contributes to TTO’s preservative potential by mitigating degradative processes in foods.

### 3.3. Antiviral Activity

The antiviral properties of tea tree oil are less extensively studied than its antibacterial and antifungal effects, but several reports indicate that TTO can inactivate or suppress certain viruses, especially enveloped viruses, at non-cytotoxic concentrations. Although antiviral studies remain comparatively limited, recent investigations suggest that TTO nanoemulsions and lipid-based nanocarriers enhance interactions between bioactive terpenes and viral envelopes, resulting in improved antiviral efficacy against several enveloped viruses. These findings indicate promising opportunities for incorporating nano-enabled TTO formulations into antimicrobial food-contact materials and sanitation systems, although further validation against foodborne viruses remains necessary. Notably, TTO has shown activity against herpes simplex viruses (HSV-1 and HSV-2), influenza A virus, and West Nile virus, among others [116]. In these studies, effective concentration is often quantified as an EC_50_ (the concentration that reduces viral infectivity by 50%). Across various experiments, TTO’s EC_50_ against HSV strains ranged from ~0.0002% up to 0.02–0.05% *v*/*v*, whereas the minimum cytotoxic concentration on host cells was around 0.025% *v*/*v*. This suggests a reasonable selective index, wherein the antiviral effect can be achieved at doses not overtly toxic to mammalian cells. Schnitzler et al. (2001) reported that TTO could reduce HSV plaque formation, with EC_50_ values for HSV-1 and HSV-2 as low as 2.0 µg/mL (0.00022% *v*/*v*) in certain assays [117]. Influenza A (H1N1) virus was also found to be susceptible to TTO: Garozzo et al. (2011) observed an EC_50_ of ~0.0006% *v*/*v* for influenza A/PR/8, while noting that host cell viability was maintained up to 0.025% TTO [118]. Similarly, a study on West Nile virus reported a 50% cytotoxic dose (CD_50_) of 0.07% *v*/*v*, implying antiviral activity at doses in that range [119].

Mechanistically, TTO’s antiviral mode of action has not been fully elucidated. Because viruses replicate inside host cells, studies have had to ensure that TTO’s effects are truly antiviral and not just due to cell toxicity [120]. Many viruses TTO is effective against are enveloped (HSV, influenza, and West Nile are all enveloped RNA or DNA viruses), suggesting that TTO might disrupt the viral envelope or interfere with envelope glycoproteins necessary for entry [121]. In support of this, TTO has a much weaker effect on non-enveloped viruses. Romeo et al. (2022) showed that tea tree oil (TTO) has notable virucidal activity against surrogate coronaviruses and may disrupt SARS-CoV-2 through multiple mechanisms [122]. A formulation containing 3.33% TTO achieved ≥99.97% inactivation of feline coronavirus after only 5 min, while human coronavirus OC43 showed 83.4% inactivation at 5 min and 95.8% after 30 min. Among the individual components, terpinen-4-ol showed the strongest activity, producing ≥99% reduction in both viruses within 5 min at 3.33%, while γ-terpinene and 1,8-cineole also showed time-dependent antiviral effects. For enveloped viruses, one might expect TTO to cause direct viral membrane damage or to alter viral attachment/entry processes. However, one detailed investigation into the influenza virus’s mechanism found that TTO did not significantly inhibit the viral neuraminidase enzyme even at 0.5%, compared to the positive control oseltamivir, nor did it prevent hemagglutination of red blood cells by the virus [118]. This suggests TTO does not block influenza binding to cells in a classical way. Instead, TTO’s antiviral effect might occur during a different stage. Some studies noted that TTO treatment reduced the yield of infectious virions and viral titers in cell culture without causing cell death, consistent with a genuine antiviral action.

In summary, while not as comprehensively characterized as its antibacterial/fungal activity, TTO’s antiviral activity appears promising, especially against viruses with lipid envelopes. The inactivation of influenza and herpesviruses by very low concentrations of TTO is a striking finding, and it has led some researchers to suggest exploring TTO for respiratory virus control or as a disinfectant [123]. Indeed, recent interest has even extended to exploring TTO against novel viruses like SARS-CoV-2 (the COVID-19 virus), due to its broad virucidal potential. γ-terpinene strongly binds to key regions of the spike protein, potentially inhibiting viral entry and fusion [122]. However, further research is needed to clarify the mechanisms, whether TTO directly dissolves viral envelopes or perhaps stimulates antiviral host responses, and to ensure safety in any such applications. It is worth noting that any in vivo or clinical use of TTO for antiviral purposes would require balancing efficacy with potential irritation or toxicity, as TTO is quite potent. Nonetheless, for surface disinfection or aerosol applications in food environments (to target norovirus, influenza on surfaces, etc.), TTO or its components might offer a natural alternative to synthetic virucides, pending more data.

### 3.4. Mechanisms of Antimicrobial Action of TTO

#### 3.4.1. Membrane Disruption and Cell Lysis

The primary mechanism by which tea tree oil exerts antimicrobial effects is through disruption of the cell membrane (and cell wall) integrity of microbes. TTO is rich in terpene hydrocarbons and related alcohols (especially terpinen-4-ol) that are highly lipophilic [96]. These molecules partition into the lipid bilayers of microbial membranes, causing increased permeability, loss of barrier function, and ultimately leakage of cellular contents. Studies by Carson et al. (2002) on *S. aureus* demonstrated that exposure to TTO or its components leads to loss of 260 nm absorbing materials (likely nucleotides and other UV-absorbing intracellular solutes) and loss of ions from the cells [124]. In *S. aureus*, 30 min of treatment with 0.25% TTO caused significant leakage of K^+^ ions and other contents, despite not immediately lysing the cells outright.

Notably, TTO-treated staphylococcal cells became markedly more sensitive to osmotic stress (NaCl), indicating they could no longer regulate their internal environment due to membrane damage. Electron microscopy of TTO- or terpinen-4-ol-treated cells shows morphological abnormalities such as mesosome-like membranous structures and loss of cytoplasmic density [125]. These are signs of cell membrane perturbation and coagulation of intracellular contents. In Gram-negative *E. coli*, similar effects have been observed, with TTO causing a rapid collapse of potassium ion gradients and, unlike in *S. aureus*, can even cause outright cell lysis in *E. coli* (especially if the outer membrane is compromised by EDTA) [126]. The greater lytic susceptibility of *E. coli* vs. *S. aureus* is thought to relate to structural differences (thinner peptidoglycan and higher inherent autolytic enzyme activity in Gram-negatives). In *P. aeruginosa*, direct mechanism studies are fewer, but it is known that *P. aeruginosa* tolerates TTO partly via its robust outer membrane; when that barrier is weakened (e.g., by Polymyxin B nonapeptide or EDTA), TTO and even less-active terpene components suddenly become much more bactericidal [127]. This underscores the membrane-centric action: any factor that eases TTO’s access to the cytoplasmic membrane (like permeabilizing the Gram-negative outer membrane) dramatically enhances its effect.

#### 3.4.2. Inhibition of Respiration and ATP Synthesis

In addition to physical membrane damage, TTO disrupts key physiological processes, such as respiration. Treated cells exhibit an inability to maintain homeostasis, reflected in arrested respiration and energy production. In *S. aureus*, TTO at MIC levels inhibits glucose-dependent respiration significantly, depriving the cell of ATP [124]. Similarly, in *E. coli*, Cox et al. (2000) noted that TTO causes immediate inhibition of oxygen consumption (respiratory activity) and that this effect is exacerbated in log-phase cells compared with stationary-phase cells [128]. The greater susceptibility of actively respiring cells suggests that TTO’s interference with membrane-bound enzymes (like those of the electron transport chain) is a critical lethal event. Indeed, the leakage of protons or collapse of ion gradients due to membrane damage will uncouple oxidative phosphorylation.

Additionally, certain TTO components might directly affect respiratory enzymes. For example, eugenol (a phenolic terpene not abundant in TTO but mechanistically like terpenoids in TTO) has been shown to inhibit mitochondrial respiration and energy production in fungi [129]. In *Candida albicans*, as mentioned, TTO caused up to a 95% reduction in respiratory rate, which would severely impair growth. This induction of a bioenergetic crisis often leads to downstream oxidative stress, as cells become unable to control electron flow; they may generate reactive oxygen species (ROS). While not extensively documented for TTO specifically, it is reasonable that treated microbes experience oxidative damage. One can infer this because loss of redox homeostasis and leaky membranes, allowing metal ions like Fe^2+^ to mislocalize, will promote Fenton reactions and ROS formation [130]. Some studies on other essential oils have detected increased intracellular ROS following terpene treatment, and organisms often upregulate antioxidant genes in response to essential oil exposure [131]. Thus, oxidative stress is likely a contributory mechanism of TTO’s antimicrobial action, secondary to the primary membrane attack.

Enzyme inhibition and cellular target disruption. Beyond membranes and respiration, TTO may inhibit various enzymes and cellular functions. The broad composition of TTO (terpinen-4-ol, α-terpineol, 1,8-cineole, etc.) means it can interact with multiple targets. For example, TTO exposure renders *S. aureus* cells autolysis-prone, not by activating autolysins directly, but by making the cell wall less stable (possibly through mild peptidoglycan hydrolase activation or interference with cell wall enzymes). Some terpenes in TTO can also inhibit membrane-bound ATPases and other enzymes. A recent study identified terpinen-4-ol as a major active component and showed it could strongly inhibit in vitro the activity of microbial spoilage enzymes such as proteases, lipases, amylases, and lactase (with ~80–90% inhibition) [132]. Such enzyme inhibition was demonstrated using TLC-separated fractions of TTO, implying a direct interaction of terpinen-4-ol with enzyme active sites or destabilization of enzyme structure. In bacteria, one known target of some essential oil constituents is the ATP synthase; inhibition of this enzyme has been reported for carvacrol and might occur with TTO components as well, contributing to ATP depletion. In summary, while membrane damage is central, TTO’s antimicrobial action likely involves a cascade of enzyme dysfunction, from dehydrogenases in the respiratory chain to hydrolytic enzymes and perhaps DNA/RNA-associated enzymes (given that cell death ultimately ensues, macromolecular synthesis must halt because of TTO treatment).

#### 3.4.3. Quorum Sensing Interference and Antibiofilm Mechanisms

An exciting aspect of tea tree oil’s bioactivity is its ability to interfere with quorum sensing (QS) signaling in bacteria, thereby attenuating virulence and biofilm formation (Table 7).

Quorum sensing, the cell-to-cell communication via small signal molecules, regulates many pathogenic behaviors (e.g., toxin production, biofilm maturation). Studies indicate that sub-lethal concentrations of TTO can disrupt QS-regulated phenomena. Noumi et al. (2018) demonstrated that TTO significantly inhibited violacein production in *Chromobacterium violaceum* (a classic QS reporter organism) by ~69% at a very low concentration (0.048 mg/mL) [135]. Violacein pigment is produced via QS; TTO’s ability to suppress it suggests interference with the *C. violaceum* QS system (likely by either scavenging the signal molecules or blocking their receptors). In the same work, TTO at 100 µg/mL (approximately 0.01% *v*/*v*) reduced the swarming motility of *Pseudomonas aeruginosa* PAO1 by one-third, and its main component, terpinen-4-ol, caused a 25% reduction in swarming. Swarming in *P. aeruginosa* is a virulence-related, QS-dependent behavior; thus, TTO clearly impairs QS-driven functions in this pathogen as well. The anti-biofilm activity of TTO and its principal constituent terpinen-4-ol has been documented against clinically and food-relevant *Staphylococcal* strains. Notably, terpinen-4-ol reduced MRSA biofilm biomass by approximately 74% at sub-MIC concentrations, an effect achieved without complete bactericidal activity and therefore attributable to interference with biofilm regulatory pathways rather than direct cell killing [136]. Mechanistically, the lipophilic terpene constituents of TTO are proposed to intercalate into the bacterial membrane and disrupt the function of membrane-associated sensor kinases and signal receptors integral to quorum sensing (QS) transduction cascades, thereby attenuating coordinated population-level responses. Evidence from broader essential oil research further indicates that certain terpene constituents can downregulate components in *Staphylococcus aureus*, a central regulatory circuit governing exoprotein secretion, virulence factor expression, and biofilm detachment, although gene-level mechanistic studies specific to TTO remain limited [137]. The phenotypic outcomes nonetheless consistently indicate QS disruption, and the convergence of membrane-targeting and regulatory interference mechanisms suggests a multi-modal basis for TTO’s anti-biofilm efficacy.

From a translational perspective, this anti-virulence activity represents a particularly valuable property for food safety applications. The capacity of sub-lethal TTO concentrations to attenuate QS-dependent coordination, suppressing biofilm establishment on processing surfaces and curtailing toxin biosynthesis, without requiring complete microbial elimination, broadens the operational dose window and reduces selective pressure for resistance development. These attributes complement TTO’s direct bactericidal activity and collectively position it as a functionally versatile antimicrobial agent suited to the complex microbial ecology of food processing environments.

#### 3.4.4. Antibiofilm Efficacy

**Figure 6:**
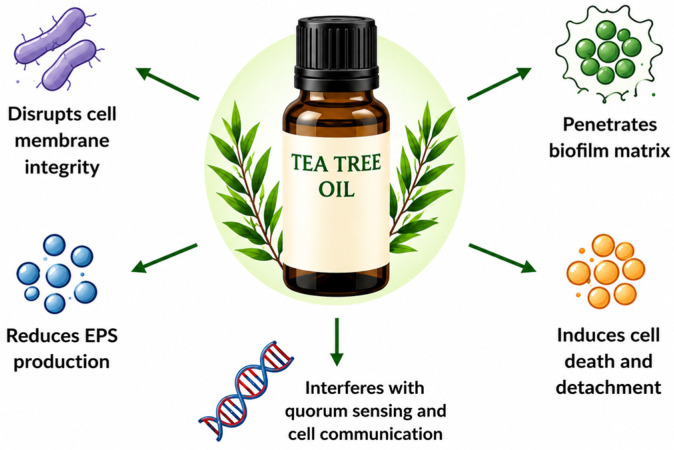
Mechanisms of antimicrobial action of TTO (created by BioRender and modified by Figure Labs).

Biofilms, structured communities of microorganisms encased in an extracellular polymeric matrix, are notoriously resistant to antimicrobials and a major concern in food processing environments, where they can harbor persistent pathogens on surfaces. The antimicrobial efficacy of tea tree oil is attributed to multiple complementary mechanisms that collectively impair microbial survival and biofilm development (Figure 6). These mechanisms include disruption of membrane integrity, inhibition of extracellular polymeric substance production, penetration of the biofilm matrix, interference with quorum sensing, and induction of cell death and biofilm detachment.

Research has increasingly addressed TTO’s effect on biofilms. Numerous studies demonstrate that although biofilm-associated cells exhibit more tolerance than planktonic cells, TTO can nonetheless inhibit biofilm formation and eliminate established biofilms at appropriate concentrations. Kwieciński et al. (2009) determined that *Staphylococcus aureus* biofilms necessitated approximately double the planktonic minimum inhibitory concentration (MIC) of tea tree oil (TTO) for elimination; however, this concentration remained below 1% *v*/*v* [138]. In their trials, around 0.5% TTO greatly diminished viable cells in a *S. aureus* biofilm, while approximately 0.25% (the MIC) was adequate to limit planktonic development but insufficient to completely remove biofilm cells. A study conducted by Brun et al. (2019) demonstrated that even sub-minimum inhibitory concentration (sub-MIC) levels of tea tree oil (TTO) can be significantly effective against MRSA biofilms, as exposure to TTO at 0.5 × MIC resulted in the death of over 70% of cells in mature MRSA biofilms [139]. The data indicate that TTO infiltrates biofilms and can provoke cell death or separation. Iseppi et al. (2020) examined the efficacy of TTO against Gram-negative biofilms composed of hospital strains of ESBL-producing *E. coli*, *Klebsiella pneumoniae*, and *P. aeruginosa* [140]. TTO, at sub-inhibitory concentrations, markedly reduced biofilm formation in a specific subgroup of these strains. For instance, 3 out of 9 *E. coli* and 5 out of 9 *Klebsiella* exhibited significant biofilm reduction following TTO therapy. While not all strains exhibited a response and *P. aeruginosa* biofilms proved more resistant, with just 2 of 9 strains demonstrating a reduction, this suggests that TTO can at least impede biofilm matrix production in certain Gram-negative bacteria. The heterogeneity underscores that changes in species and strain-level biofilm matrix composition (and potentially efflux activity) affect TTO performance.

TTO’s antibiofilm action extends to fungal biofilms as well. *Candida albicans* biofilms on surfaces (like dentures or medical devices) pose a challenge, but TTO has shown potency here too. Soaking denture acrylic in 1% TTO for just 5 min resulted in a significant reduction in *C. albicans* biofilm mass [141]. Comparatively, mature bacterial biofilms can be more stubborn. Liu et al. (2020) reported that *S. aureus* and *E. coli* biofilms on medical-grade surfaces required up to 8% TTO to destroy 50% of the biofilm, indicating a higher threshold for disruption [142]. Such concentrations may be impractical for direct use, but they spurred investigations into TTO delivery systems to enhance antibiofilm performance. For instance, researchers have encapsulated TTO in nanoparticles or cyclodextrin complexes to increase its stability and penetration. Casarin et al. (2019) conducted a clinical trial comparing tea tree oil to chlorhexidine (0.12%) for disrupting dental plaque biofilms [143]. The TTO nano-formulation did reduce plaque indices, though it was somewhat less effective than chlorhexidine at removing established biofilm and was less palatable to participants. Nonetheless, this exemplifies how TTO can be harnessed in controlled-release formats to combat biofilms.

#### 3.4.5. Relevance to Food Safety

Biofilms in food industries (e.g., on cutting machines, piping, food storage tanks) often involve mixed communities of bacteria and fungi that protect pathogens from cleaning agents. TTO’s ability to both kill planktonic cells and penetrate biofilms means it could play a role in surface sanitation or food-contact surface coatings.

Tea tree oil (TTO) and inorganic nanoparticles such as ZIF-8 exhibit distinct yet complementary antibiofilm mechanisms. TTO primarily acts through its lipophilic terpenes (e.g., terpinen-4-ol), which penetrate and disrupt microbial cell membranes, increase permeability, and interfere with quorum sensing, thereby inhibiting biofilm formation and promoting biofilm dispersal [144]. However, its efficacy can be limited by volatility, instability, and reduced activity in complex matrices. In contrast, ZIF-8-based nanoparticles provide a more stable and tunable platform for antibiofilm control. Their mechanisms include sustained release of metal ions (e.g., Zn^2+^), generation of reactive oxygen species (ROS), and direct interaction with extracellular polymeric substances (EPS), leading to structural disruption of established biofilms. Moreover, ZIF-8 can serve as a carrier for bioactive compounds like essential oils, enabling controlled release and enhanced penetration into biofilm matrices [145].

Some studies have explored TTO in coatings or films. For example, chitosan films with TTO droplets showed sustained antimicrobial effects on surfaces. Cai et al. (2024) developed a chitosan-modified self-nanoemulsion (TNE) encapsulating TTO to enhance stability and efficacy [146]. The formulation (212 nm size, low PDI, −20.5 mV zeta potential) demonstrated improved physicochemical stability, biocompatibility, and 2–8-fold stronger antibacterial activity than conventional suspensions. In vivo, TNE reduced inflammation, promoted ulcer and wound healing, and disrupted bacterial metabolism by altering protein expression. Another group created mesoporous silica loaded with TTO, achieving a long-lasting antibacterial surface that slowly releases TTO vapors [24]. These innovations are aimed at overcoming the volatility of TTO and ensuring it remains at effective concentrations over time.

Lastly, it is worth noting that TTO’s efficacy against biofilms has been validated not just in vitro but also in animal models. An immunosuppressed mouse model of *Candida* oral infection (thrush) was used by De Campos Rasteiro et al. (2014) to test TTO [147]. In vitro, they found the *Candida* MIC was 0.195%, but a 65 × higher concentration (12.5% TTO) was needed to fully eradicate the biofilm, underscoring how tough biofilms are. When applying TTO treatment in the infected mice, a notable reduction (~5-fold) in fungal load (CFU from tongue swabs) was achieved, although it did not eliminate lesions. This suggests timing and delivery are critical; frequent or early application might be required for full biofilm clearance in vivo. Other in vivo infection models (vaginal candidiasis, oral infections, even a viral encephalitis model for West Nile) have been explored with TTO with some positive outcomes [148].

**Figure 7:**
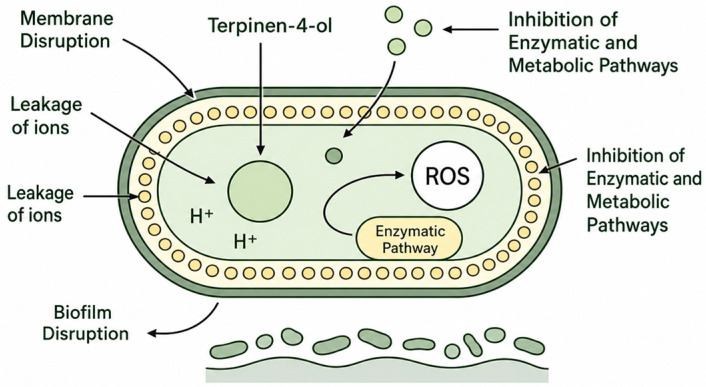
Mechanisms of antimicrobial action of tea tree oil (created by BioRender and modified by Figure Labs).

In conclusion, tea tree essential oil demonstrates significant antimicrobial and antibiofilm activities relevant to food safety. It can inhibit a wide array of foodborne bacteria and fungi, disrupt quorum sensing and biofilm formation (thus reducing pathogen persistence and virulence), and even inactivate certain viruses. Its mechanisms, principally membrane disruption, but also induction of oxidative stress, enzyme inhibition, and signaling interference, make it a multifaceted antimicrobial (Figure 7).

TTO’s performance is often on par with or complementary to other potent essential oils (like oregano, thyme, or clove). While practical use in foods may be limited by sensory considerations at higher doses, strategies like encapsulation, vapor-phase application, or combining sub-lethal TTO with other hurdles (e.g., mild heat or low pH) could leverage its antimicrobial power without adverse effects on food quality [149]. Ongoing research into the formulation and delivery of TTO aims to overcome these challenges, opening the door for TTO to be utilized as a natural preservative and surface sanitizer in the food industry [150].

## 4. Applications of Tea Tree Essential Oils in Food Safety

Tea tree essential oil (TTO) has attracted increasing interest as a natural preservative in various food systems due to its broad-spectrum antimicrobial and antifungal activities. TTO’s major components (e.g., terpinen-4-ol, α-terpineol, 1,8-cineole) confer potent effects against foodborne bacteria, molds, and yeasts [151]. From a regulatory standpoint, TTO is currently sanctioned solely as a flavoring substance for indirect or trace-level use; it is not approved as a direct antimicrobial additive or preservative at the concentrations required for meaningful food safety intervention [152]. This distinction is further underscored by the U.S. Food and Drug Administration’s 2019 final rule [152], which explicitly excludes TTO from recognition as an over-the-counter antiseptic agent. Nevertheless, the direct incorporation of TTO into food matrices presents substantive technological challenges, including its pronounced organoleptic impact, high volatility, and poor aqueous solubility [150]. To address these limitations, considerable research effort has been directed toward advanced delivery systems, encompassing nanoemulsions, microencapsulation, edible coatings, and active packaging, designed to preserve antimicrobial efficacy while mitigating sensory and physicochemical constraints [149]. This section provides a systematic review of TTO applications across key food categories (meat, dairy, seafood, fresh produce, and beverages), examines the enabling technologies facilitating its use, and discusses relevant case studies and functional applications related to food safety.

### 4.1. Meat and Poultry Products

**Figure 8:**
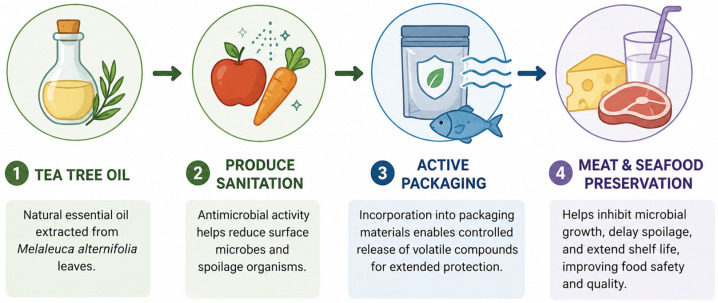
Tea tree oil application process in meat and seafood preservation (created by BioRender and modified by Figure Labs).

Meat and poultry are highly perishable and prone to contamination by pathogens (e.g., *Listeria monocytogenes*, *Salmonella*, *Escherichia coli*) and spoilage microbes. Tea tree oil has been studied as a natural antimicrobial to extend the shelf life and safety of these products (Figure 8).

In ground beef, TTO demonstrated remarkable antilisterial activity. At 1.5% (*v*/*w*) concentration, it caused a rapid drop of *L. monocytogenes* by ~5 log CFU/g within 20 min [94]. This dramatic bactericidal effect in meat matrices highlights TTO’s potential to control lethal pathogens. Notably, the efficacy was influenced by the initial contamination level; TTO treatments were fully effective up to moderate inoculum sizes (≤5 log CFU/g), whereas extremely high loads (~8 log CFU/g) were more difficult to eliminate. Still, across realistic contamination levels in foods, TTO exhibits significant antimicrobial action.

TTO’s ability to preserve meat quality has been confirmed in recent shelf-life studies. Moirangthem et al. (2024) reported that incorporating 1% tea tree essential oil in raw chicken fillets markedly delayed microbial spoilage and oxidative deterioration during refrigerated storage [62]. Treated fillets showed lower total viable counts and reduced lipid oxidation compared to controls, resulting in an extension of shelf-life by up to 7 days over untreated samples. Impressively, the TTO-treated chicken maintained better color and odor, indicating that the oil’s antimicrobial action prevented the development of spoilage off-odors rather than introducing undesirable aromas [153]. Similarly, a combination of tea tree and nutmeg oils (each at 1%) was found to preserve the quality of chicken meat, reinforcing that TTO can serve as a natural preservative to enhance meat safety and freshness [62].

Beyond laboratory experiments, researchers have integrated TTO into novel meat packaging systems. One approach is active packaging films that slowly release the oil’s vapors to continuously inhibit microbial growth on the product’s surface. Liu et al. (2025) developed a chitosan-based film using a Pickering emulsion loaded with tea tree oil for pork preservation [154]. This advanced film exhibited a controlled release of TTO and strong adhesion, which together prevented rapid evaporation of the oil and prolonged its antimicrobial effect on stored pork. TTO significantly suppressed bacterial growth in pork and maintained the meat’s quality over extended cold storage, demonstrating how packaging technology can apply TTO in a practical, real-world manner [155]. Such active packaging could be especially valuable for ready-to-eat or fresh meats where surface contamination is a concern.

It is worth noting that while TTO is effective, its intense flavor can potentially affect meat sensory properties if used in high doses. Overall, the evidence indicates that TTO can extend the shelf-life of meat and poultry, lower pathogen counts and maintain sensory acceptability when applied appropriately. Incorporating TTO via marinades, coating sprays, or active packaging is a viable strategy to harness its preservative benefits in the meat industry.

### 4.2. Dairy Products and Cheese

Applying tea tree oil in dairy products (milk, cheese, etc.) is challenging due to the delicate flavors of these foods and the oil’s strong taste. Nonetheless, researchers have explored TTO as a natural agent to combat dairy spoilage microbes and pathogens. In cheese production, plant essential oils can inhibit mold growth and pathogenic bacteria, potentially reducing reliance on synthetic preservatives. Tea tree oil has shown antimicrobial activity in soft cheeses, but often only at relatively high concentrations that risk altering flavor. For example, Selim (2011) tested TTO in feta cheese contaminated with *Escherichia coli* O157:H7 and vancomycin-resistant enterococci and observed significant microbial inhibition only at 0.5–1% oil levels [110]. Unfortunately, at those levels the cheese developed a pronounced off-flavor. Clove oil displayed a similar issue in the same study, whereas a smaller amount of thyme oil was effective with less sensory impact. This illustrates a key hurdle that the concentrations of TTO needed for robust antimicrobial action in dairy can exceed sensory thresholds, leading to unacceptable medicinal or herbal notes in the product.

To address this, recent work has focused on incorporating tea tree oil into coatings or packaging for cheeses, rather than direct mixing into the curd. Edible antimicrobial coatings (e.g., whey protein or polysaccharide films infused with TTO) can be applied to cheese surfaces to suppress mold growth during ripening [156]. Such coatings create a barrier that gradually releases the oil’s volatiles in situ. Although specific data on TTO-edible films for cheese are limited, analogous essential oils (like oregano or cinnamon oil coatings) have successfully extended cheese shelf-life by inhibiting surface microbial growth. By the same principle, a tea tree oil coating could prevent mold spoilage on cheese rinds without substantially migrating into the cheese interior, thus minimizing flavor taint.

Another promising avenue is active packaging sachets for dairy. A small sachet containing an essential oil-infused absorber could be placed inside cheese packaging to emit antimicrobial vapors into the headspace [157]. This approach, akin to desiccant packs, avoids direct oil-food contact. One study reported that integrating 0.2% tea tree oil into a gelatin-based film significantly reduced mold growth on packaged mushrooms, suggesting similar efficacy might be achieved in dairy contexts [158]. In fluid dairy (milk, cream), TTO is rarely used because it is hydrophobic and would need emulsifiers to disperse. Moreover, raw milk already has natural antimicrobial enzymes, such as lactoperoxidase; adding a strong essential oil could disrupt fermentation or flavor of cultured products [159]. However, for certain high-risk dairy foods (e.g., queso fresco or raw milk cheese), TTO could serve as a surface sanitizer. A brief exposure to dilute TTO spray on cheese surfaces might reduce listerial contamination without significant sensory uptake, but more research is needed.

In summary, tea tree oil’s antimicrobial efficacy in dairy is recognized, but its practical use requires careful formulation. High concentrations ensure microbial safety but can introduce sensory defects. Current strategies thus emphasize indirect application, e.g., TTO in coatings, films, or packaging, to protect dairy products from spoilage and pathogens while keeping the oil’s flavor impact to a minimum. These technologies are still largely at experimental stages, and further pilot-scale studies will be crucial to determine consumer acceptance of TTO-protected dairy foods.

### 4.3. Seafood and Fish Products

Seafood is another category where tea tree essential oil has shown considerable promise in enhancing food safety. Fish and shellfish are highly perishable due to rapid microbial spoilage (from *Pseudomonas*, *Shewanella*, *Lactic acid bacteria*, etc.) and lipid oxidation, especially under refrigeration [110]. Essential oils are being explored as natural alternatives to synthetic ice glazes or sulfur-based preservatives in this sector. TTO exhibits both antibacterial and antioxidant properties that can be leveraged to keep seafood fresh for longer.

One innovative application is the use of antimicrobial nanofiber coatings for fresh fish fillets. Xia et al. (2023) developed an electrospun chitosan-based nanofiber mat loaded with tea tree oil for wrapping fresh salmon fillets [113]. The ultrafine fibers (∼200 nm diameter) act as a breathable, edible layer on the fish surface, continually releasing small amounts of TTO during storage. This approach yielded impressive results with the TTO-infused nanofiber coating delaying spoilage and significantly extending the shelf life of salmon fillets during chilled storage. Treated fillets showed lower microbial counts (total viable count), slower increases in thiobarbituric acid (TBA) values (indicating reduced oxidative rancidity), and better texture and color retention compared to uncoated controls [160]. By sensory evaluation, salmon wrapped in the TTO nanofiber remained acceptable for several days longer than untreated fish, demonstrating a clear shelf-life extension. This study highlights how embedding tea tree oil in a stabilizing matrix (chitosan nanofibers) can overcome the oil’s volatility and achieve a controlled, long-lasting antimicrobial effect in seafood packaging.

Tea tree oil has also been examined as part of edible coatings for fish. For instance, a gelatin-based edible coating incorporating TTO was tested on fresh trout fillets and found to inhibit bacterial spoilage while maintaining the fish’s natural flavor [161]. The coating reduced the growth of common spoilage bacteria, leading to approximately a 5-day extension of shelf life at 4 °C (from ~2–3 days for control fish to ~7–8 days for coated fish, as judged by sensory acceptability and microbial limits). In another approach, researchers have used modified atmosphere packaging (MAP) combined with slow-release TTO formulations. By adsorbing tea tree oil onto inorganic carriers (like nano-silica) and then embedding it in a starch-based matrix, a solid slow-release preservative can be made [162]. Lin et al. (2018) applied such a sustained-release TTO formulation to fresh-cut seafood and fresh-cut fruit, demonstrating its effectiveness and suggesting it could similarly be applied to fish fillets [163]. A starch/carboxymethylcellulose film with nano-dispersed organic nanoparticles, such as tea tree oil, could translate to fish, creating a moisture-resistant coating that both protects against microbial growth and oxidative spoilage [164].

Active packaging for seafood is another emerging area. In one case, a prototype polypropylene film was impregnated with microcapsules of essential oils and used to package shrimp; the active film significantly reduced microbial load on the shrimp and delayed the onset of spoilage odors compared to regular film over a week of storage [165]. Likewise, for chilled carp fillets, fumigation with small amounts of tea tree oil in the package headspace has been reported to suppress surface bacterial growth and improve sensory scores, reducing fishy odor development [166]. These real-world simulations indicate that TTO can be harnessed in seafood supply chains, for example, by including a TTO-emitting pad in fish boxes or by misting fish with a dilute TTO nanoemulsion before sealing in ice.

It is important to note that the flavor of fish is generally robust enough that a slight tea tree note (often described as camphoraceous or herbal) might not be objectionable if kept subtle. Consumer tests on TTO-treated fish are still limited, but preliminary observations suggest that when TTO is controlled-release (as in nanofibers or microcapsules), the fish does not develop any distinctly off-putting flavor. In summary, through modern delivery systems, tea tree oil can extend the freshness of seafood by curbing microbial spoilage and oxidative changes, with studies reporting several days of extra shelf-life and improved microbiological safety for treated fish products.

### 4.4. Fruits, Vegetables, and Fresh Produce

Fresh produce (fruits, vegetables, herbs) often has a short post-harvest life due to decay caused by fungi and bacteria. Tea tree essential oil, with known antifungal potency, has been widely investigated as a natural post-harvest treatment to reduce spoilage and ensure the safety of fruits and fresh produce [167]. Unlike foods that are cooked, produce is frequently consumed raw, so controlling pathogens (like *E. coli* or *Salmonella* from contamination) on surfaces is also a critical food safety challenge. TTO has demonstrated efficacy in both reducing post-harvest diseases (molds, rots) and inactivating human pathogens on produce surfaces.

#### 4.4.1. Applications in Fruits

TTO is particularly effective against a range of fruit pathogens and spoilage fungi. One prominent example is its action against *Botrytis cinerea*, the gray mold responsible for major losses in berries and grapes. Liu et al. (2025) developed a high-adhesion composite edible coating using quaternized chitosan (HACC) conjugated with oleic acid as a carrier for TTO, aimed at strawberry preservation [154]. The amphiphilic HACC-oleate matrix improved the spreading and retention of the coating on strawberry surfaces and significantly slowed TTO evaporation (only ~27.8% lost over a set period, versus ~49.5% loss for pure TTO. This coating showed synergistic antifungal activity that lowered the effective concentration needed to inhibit *B. cinerea* compared to chitosan alone. In practical terms, strawberries coated with the TTO-HACC formulation had much lower mold growth and decay index during storage. The treated fruits maintained their bright color and firmness, had less weight loss, a slower decline in vitamin C, and overall appeared fresher than untreated berries. By delaying mold spoilage and preserving texture, the TTO coating substantially extended the strawberries’ shelf-life. This study underscores how edible coatings carrying tea tree oil can protect fresh fruit quality, leverage TTO’s antifungal power while mitigating its volatility.

Another approach for fruits is fumigation or vapor-phase application of tea tree oil [168]. Because TTO is rich in volatile compounds, simply enclosing fruits in a container with a small amount of TTO can impart antimicrobial effects in the gaseous phase. Researchers have found that TTO vapor can reduce the incidence of common fruit diseases. For example, citrus fruits stored in bins with tea tree oil vapors showed reduced green mold (*Penicillium digitatum*) development, and berries exposed to TTO vapor combined with hot air had lower *Botrytis* infection rates than controls [169,170]. Tea tree oil’s volatility is a double-edged sword as it allows easy delivery as a gas, but it can dissipate quickly. Thus, sustained-release systems are preferable. The use of β-cyclodextrin inclusion complexes or encapsulated TTO in sachets has proven effective in slowly releasing the oil’s vapors over time in produce packaging.

#### 4.4.2. Applications in Vegetables and Fresh Cuts

Tea tree oil has shown benefits in preserving minimally processed vegetables and fresh-cut produce as well. Guan et al. (2024) evaluated TTO on lightly processed Lanzhou lily bulb scales, a vegetable used in Asian cuisine, which tend to discolor and spoil after peeling [171]. Treatments with low doses of TTO (25–100 µL/L) helped delay quality deterioration: TTO significantly slowed weight loss, maintained firmness and visual appeal, and suppressed microbial growth on the lily bulbs. The optimal dose (50 µL/L) preserved the best sensory quality and microbiological safety during storage. TTO also induced the vegetable’s own defense responses that increased phenolic content and antioxidant enzyme activities (like SOD, APX) in the plant tissue, which helped mitigate oxidative browning and spoilage. At the same time, TTO inhibited enzymes like lipoxygenase that lead to lipid peroxidation, thereby reducing membrane damage and malondialdehyde accumulation. These findings suggest that TTO not only directly kills contaminants but also activates plant defense mechanisms and slows senescence in fresh-cut produce. The net effect was prolonged shelf-life and improved retention of nutritional quality (e.g., vitamin C, phenolics) in the treated lily bulbs.

For fresh-cut fruits such as pineapple, TTO-based preservatives have been used in combination with modified-atmosphere packaging. In a study of Tian et al. (2023), a sustained-release tea tree oil solid preservative was formulated by adsorbing TTO on nano-silica and embedding it in a starch/carboxymethylcellulose matrix [172]. When a small packet of this solid was placed with fresh-cut pineapple chunks in an MA-pack, it continuously released TTO vapor in situ. The results were notable, as the tea tree oil preservative significantly improved the sensory quality and reduced microbial spoilage of the cut pineapple. Treated pineapple pieces remained more brightly colored, firmer, and had lower microbial counts (total plate count and yeast/mold) over 4 days of storage, whereas control samples quickly fermented and discolored. In fact, the TTO treatment approximately doubled the shelf-life of the fresh-cut pineapple to 4 days, compared to about 2 days for untreated fruit under the same conditions. Moreover, the pineapple retained higher levels of antioxidants, and enzymes like peroxidase and polyphenol oxidase were modulated favorably, correlating with slower quality degradation. This example demonstrates the feasibility of using TTO in a commercial post-harvest setting, a sachet or coating that can be easily included in produce packaging to naturally prolong freshness.

Across diverse produce items (strawberries, citrus, cut fruits, leafy greens, etc.), tea tree oil has proven effective in reducing spoilage, disease incidence, and pathogen loads. It can be applied via dipping, spraying, waxing, coating, or volatilization, often in combination with other hurdles (refrigeration, modified atmospheres) for synergistic preservation. Importantly, studies frequently note that TTO treatments maintained or even improved sensory attributes of produce [173]. By preventing decay, TTO keeps produce looking and tasting fresh; and when properly formulated, its herbal scent is mild and well-masked by the natural aromas of the fruit or vegetable. Given the growing demand for residue-free, green post-harvest solutions, tea tree oil emerges as a promising candidate to enhance the safety and shelf-life of fresh produce in an eco-friendly manner.

### 4.5. Beverages and Liquid Food Systems

Using tea tree essential oil in beverages is less common than in solid foods, mainly due to the challenges of flavor compatibility and dispersibility. TTO has a strong, medicinal taste that can be off-putting if it remains in the final drink. However, in principle, its antimicrobial activity could be beneficial in certain beverage contexts. For example, as a natural preservative in fruit juices, smoothies, or functional drinks that are prone to microbial spoilage. A few studies have tested TTO in model beverage systems to assess its efficacy. TTO was added to fresh cucumber juice as a representative high-pH vegetable juice, which can support pathogenic bacteria like *Listeria monocytogenes* [96]. Remarkably, at a concentration of 2% *v*/*v*, TTO completely inhibited *L. monocytogenes* in the juice, achieving bactericidal effects within 12 h at both refrigeration temperature (4 °C) and room temperature (25 °C). This indicates that TTO’s antimicrobial constituents remain active in an aqueous juice matrix, and that their effect is not strongly temperature-dependent in the short term. By contrast, a much lower dose (0.25% TTO) was insufficient to halt *Listeria* growth at 25 °C, with no inhibition even at 48 h, though interestingly it still achieved about 90% killing at 4 °C after 2 days. These results suggest that a threshold concentration of TTO is required for preservative efficacy in liquids, and that at sub-lethal levels some bacteria might survive, especially at abuse temperatures. Still, the potent anti-*Listeria* activity at 2% underscores TTO’s potential as a natural sanitizer for juices or other beverages, if concentration can be tolerated in terms of flavor.

To overcome the solubility issue in drinks, as oils do not mix with water, TTO would likely be incorporated via emulsification. Nanoemulsion technology is particularly relevant here. By creating ultra-fine emulsions of tea tree oil (droplet sizes on the order of 50–100 nm), the oil can be dispersed uniformly through a liquid, and its bioactivity can be enhanced. Cen et al. (2025) showed that formulating TTO into a carboxymethyl chitosan/Tween 80 nanoemulsion not only improved its stability in aqueous environments but also significantly enhanced its antimicrobial efficacy as measured by lower minimum inhibitory concentrations (MICs) against foodborne bacteria [9]. The TTO nanoemulsion had a much stronger antibacterial effect than non-emulsified (bulk) TTO in laboratory media, presumably because the tiny oil droplets increased contact with microbial cells and possibly facilitated uptake of TTO components. Additionally, the nanoemulsified TTO showed higher antioxidant activity than bulk oil, which could help prevent oxidative spoilage in beverages [174]. These findings imply that if TTO were to be added to a beverage (such as fruit juice or a nutraceutical drink), using a nanoemulsion would maximize its preservative function while minimizing problems like phase separation. Some commercial beverage preservative systems use essential oil nanoemulsions (e.g., of citrus or thyme oils). A similar approach with tea tree oil could yield a natural antimicrobial beverage additive that extends shelf-life and ensures microbial safety in products like unpasteurized juices or kombucha [175].

That said, the flavor impact remains a concern. Most consumers would not expect a tea tree flavor in their juice or beer. One way to circumvent this is to use TTO in combination with strong-flavored beverages or ingredients that could mask its taste [176]. For example, a spiced health tonic or a botanical beverage might incorporate a very small amount of tea tree oil alongside mint, ginger, or other pungent flavors, creating a complementary profile. TTO is sometimes described as having notes like medicinal eucalyptus; in tiny quantities, it might blend into minty or herbal drinks. Another application could be in syrups or concentrates that are diluted for consumption—the concentrate (with TTO) would be self-preserving, but upon dilution, the TTO level might fall below taste perception.

In summary, while tea tree essential oil is not widely used in mainstream beverages due to organoleptic issues, it can act as a powerful natural preservative in liquid foods. Research demonstrates that TTO can inactivate pathogens in juices and likely inhibit spoilage microbes (yeasts, lactic acid bacteria) that cause fermentation of beverages. The key to practical use will be advanced formulations (nanoemulsions, microencapsulation) to disperse the oil and pair with flavors that tolerate its presence. At present, TTO’s role in beverages is mostly experimental, but it could find niche applications in the future for functional drinks or specialty juices where natural preservation is paramount, and flavor profiles are adjustable.

### 4.6. Delivery Systems for TTO in Food Applications

Because of tea tree oil’s volatility, poor water-solubility, and intense aroma, delivery systems play a crucial role in applying TTO effectively in foods. Recent advances in food technology have yielded various methods to encapsulate or incorporate TTO in a controlled manner, thereby enhancing its stability and antimicrobial performance while reducing sensory impact. Key delivery approaches include nanoemulsions, microencapsulation, edible coatings, and active packaging. These systems often make the difference between theoretical antimicrobial and practical food preservative.

#### 4.6.1. Encapsulation Technologies for Enhanced Stability and Controlled Delivery of TTO

Nanoemulsions are ultra-fine oil-in-water emulsions that can carry essential oils like TTO in the form of tiny droplets (10–100 nm) [177]. By dramatically increasing the oil’s surface area and dispersion in aqueous media, nanoemulsification improves TTO’s contact with microbes and its chemical stability. In practical terms, this means nanoemulsion could be mixed into a food or coating solution and remain effective over the product’s shelf-life. Another benefit observed was improved antioxidant activity of TTO after nanoemulsification, which can help protect foods from oxidative rancidity in addition to microbial spoilage. Given these advantages, nanoemulsion technology is considered a promising strategy to incorporate tea tree oil into food systems. For example, a nanoemulsified TTO could be sprayed onto produce or cooked meats as a disinfectant mist, or included in a beverage as discussed, with far less risk of phase separation or flavor pockets than crude oil [178].

Microencapsulation is another technique, wherein TTO droplets are enclosed in protective wall material (e.g., polysaccharides, proteins, or lipids), forming microcapsules or microspheres. Microencapsulation can markedly reduce the volatility of tea tree oil and enable controlled release of its active components over time. Han et al. (2025) prepared TTO microcapsules using β-cyclodextrin (a cyclic starch) combined with nano-montmorillonite clay as the encapsulating matrix [179]. The resulting microcapsules had an encapsulation efficiency of ~78% and greatly slowed the evaporation of TTO compared to the free oil (at 60 °C and 90 °C, the capsules released significantly less TTO vapor). When these microcapsules were placed with strawberries in storage, they continuously emitted small amounts of TTO, enough to suppress fungal decay and quality loss. The optimal dose was 5 g of microcapsules per 1.2 L of storage space, which effectively preserved strawberries for over two weeks with minimal spoilage. This illustrates how microencapsulation enables a time-release preservative effect as the oil is released gradually from the capsule matrix, maintaining an antimicrobial atmosphere around the food for an extended duration. Cyclodextrin inclusion complexes of TTO have similarly been reported to retain around 60–70% of the oil after a month of storage (unencapsulated oil would largely evaporate in that time) [179]. Thus, microencapsulation not only improves the shelf stability of the oil itself but also solves the problem of rapid dissipation, making TTO’s action more sustained during food storage.

Various encapsulation materials have been tried. Polysaccharide-based capsules (like the starch/CMC/nano-silica system in the pineapple study) provide a solid reservoir for TTO and can be incorporated into packaging or coatings easily [180]. Lipid-based capsules or emulsions can protect TTO from oxidation and release it when the lipid matrix melts or is digested (potentially useful for internal release in the gut if designing functional foods). Even protein matrices (gelatin, whey protein) have been used to encapsulate TTO and other essential oils for controlled release on fresh produce. The choice of encapsulation method depends on the intended application: for instance, cyclodextrin complexes are excellent for dry applications (powders, sachets), while nanoemulsions are ideal for liquid applications (sprays, dips). Sanchez-Navarro et al. (2011) explored tea tree oil as a natural antimicrobial agent for footwear applications [181]. Microencapsulation using melamine-formaldehyde enhances its stability against moisture, light, and oxygen, improving durability.

In summary, nanoemulsification and microencapsulation are critical enabling technologies that allow the effective use of tea tree oil in foods. They enhance antimicrobial efficacy, protect the oil from premature evaporation or degradation, and mitigate sensory impact by slowing release (a slow release means the concentration of TTO in the food at any given time stays low, avoiding overpowering flavor while still suppressing microbes). These techniques bridge the gap between TTO’s impressive lab results and its practical functionality in real food systems.

#### 4.6.2. Edible Coatings and Films

Edible coatings and films refer to consumable wraps or surface layers applied to foods, which can be formulated with antimicrobial agents. Tea tree oil has been successfully incorporated into a variety of edible coating materials to create active films that protect food surfaces [182]. The benefit of coating is that it localizes the antimicrobial where it is most needed (the food surface and the surrounding microenvironment) and acts as a barrier to moisture and gases, often improving the food’s overall storage stability.

Common edible coating bases include polysaccharides (like chitosan, alginate, pectin), proteins (gelatin, whey protein), and lipids (beeswax, fatty acid compounds). TTO can be emulsified into these bases. Chitosan has received attention because it innated antimicrobial properties and forms good films. When tea tree oil is added to chitosan, the combination can yield a synergistic effect [183]. For example, a chitosan coating with TTO was shown to more effectively inhibit mold on oranges and strawberries compared to chitosan alone. The film-forming ability of chitosan helps to evenly distribute TTO on the fruit, while TTO broadens the spectrum of antimicrobial activity. Similarly, gelatin films containing tea tree oil have been used on fresh-cut produce and fish, reducing microbial loads and delaying quality loss [184].

One challenge with incorporating essential oils into hydrophilic edible films is achieving good dispersion of the oil and preventing it from leaking out. Recent advances in edible coating technologies have increasingly focused on nanoemulsion-based and encapsulated delivery systems to improve the dispersion, stability, and controlled release of essential oils within biopolymer matrices, thereby enhancing antimicrobial efficacy while minimizing undesirable sensory effects [6]. Techniques like forming oil-in-water emulsions before mixing into film solutions are employed. Alternatively, Pickering emulsion films have been developed, where solid particles (e.g., cellulose nanocrystals, chitin nanoparticles) stabilize the oil droplets in the film matrix. Another interesting edible film is one made from starch or derivatives. Starch-based films can incorporate TTO either by direct emulsion or via inclusion complexes. As noted with the pineapple example, a starch/CMC matrix successfully delivered TTO vapors in a fresh-cut fruit package. Likewise, researchers have made whey protein isolate films with tea tree oil aimed at cheese preservation, with the protein matrix helping to trap TTO and release it slowly to inhibit surface mold, although high oil content could weaken the film unless plasticizers were adjusted [156].

In edible coating applications, sensory and appearance factors are important. A good coating should dry to a thin, invisible layer without a sticky or greasy feeling. Incorporating TTO at 0.5–1.0% usually does not visibly alter the film, though higher amounts can cause strong odors or a cloudy appearance. The high-adhesion chitosan coating by Liu et al. (2025) showed that modifying chitosan to be more hydrophobic (via oleic acid grafting) improved its film uniformity on fruit and helped retain TTO [154].

This resulted in no white residue on the strawberries and a pleasant appearance. Additionally, consumers could not distinctly detect tea tree oil on coated fruits in informal taste tests, suggesting that if the oil is encapsulated in the coating and used at effective yet low concentrations, it will not necessarily impart a noticeable flavor. In summary, edible films and coatings are a versatile and consumer-friendly means of applying TTO to foods. They are especially useful for commodities that are sold as fresh or minimally processed (fruits, vegetables, cheeses, meats) where direct addition of liquid preservatives is not feasible. By tailoring film composition and using emulsification techniques, manufacturers can create coatings that prolong shelf-life and food safety through TTO’s antimicrobial action without compromising the food’s natural appeal.

#### 4.6.3. Active Packaging Technologies

Active packaging refers to packaging systems that do more than passively contain the food; they actively interact to improve food quality or safety. TTO has been employed in active packaging in several forms, from emitter pads to antimicrobial plastic films. The main idea is to integrate tea tree oil into the packaging material so that it continuously releases antimicrobial vapors into the food’s environment during storage.

One approach, as mentioned, is embedding microcapsules of tea tree oil into packaging films. Researchers have incorporated TTO-loaded microcapsules in bioplastic films (e.g., polylactic acid or starch-based plastics) [164]. These films slowly emit tea tree oil and can reduce surface contamination on foods they wrap. For instance, a starch-based active film with a covalently bound framework to hold tea tree oil was designed to have improved oil retention. This film demonstrated sustained antibacterial activity, though detailed results are proprietary in some cases.

A notable success was reported in the packaging of fresh mushrooms (*Agaricus bisporus*). A polyvinyl alcohol/sorbitol film containing essential oil was developed that, when used to wrap mushrooms, significantly slowed microbial spoilage and browning, extending shelf-life by a few days compared to conventional packaging [185]. The film also reduced the mushroom’s respiration rate due to its partial barrier properties, further prolonging freshness.

In active modified-atmosphere packs, tea tree oil can be included as part of an atmosphere conditioning system. A sachet with TTO solid preservative in an MA package of pineapple was effective in inhibiting bacteria [54]. Similarly, such a sachet could be included in salad bags or deli meat packages to suppress *Listeria* and other psychrotrophic bacteria. A recent review noted that plant essential oil emitters in salad bags could achieve 1–3 log reductions in *L. monocytogenes* on inoculated produce during refrigerated storage. Tea tree oil, being highly volatile, is well-suited for such vapor-phase applications. Its vapors can penetrate the nooks of complex foods like salads better than liquid washes.

Active packaging with TTO has also been proposed for bakery products. Although not a food safety issue in terms of pathogens, mold growth on bread and cakes is a major spoilage problem. A TTO-emitting packaging (for instance, a paper insert infused with TTO) can release antifungal vapor in a bread box, preventing mold spores from germinating on the product [186]. Trials have shown that a low dose of tea tree oil vapor can prolong bread mold-free time by several days, though care is needed that the bread does not absorb a eucalyptus-like aroma.

From an industrial perspective, active packaging with natural essential oils like TTO is attractive because it can be implemented relatively easily (e.g., by adding oil or capsules during film extrusion or including a small pad in the package) and does not require direct contact of additives with the food [187]. Regulatory-wise, the packaging is considered the carrier, and if the essential oil is GRAS and used in small quantities, it can be acceptable. Companies are indeed experimenting with essential-oil active packaging as a selling point for chemical-free preservation. Tea tree oil’s unique antimicrobial spectrum (effective against bacteria and fungi) makes it a strong candidate for such uses.

In conclusion, delivery systems ranging from nanotech encapsulations to active packaging greatly expand the practicality of using tea tree essential oil in food safety applications. They address the inherent challenges of using volatile oil in complex food matrices and pave the way for commercialization of TTO-based preservative solutions.

### 4.7. Industrial and Real-World Case Studies

While much of the research on tea tree oil in food safety remains at the laboratory or pilot scale, there are emerging examples of real-world applications and commercial interest. Here, we highlight some case studies and practical considerations of using TTO in food industry settings.

One case study is the use of TTO in a commercial fresh-cut produce operation. Building on academic findings, a produce processor conducted a pilot trial where fresh-cut lettuce was packaged in plastic clamshells with a small filter paper impregnated with tea tree oil. The goal was to reduce microbial load and spoilage in packaged salads [188]. The trial found that the tea tree oil pad reduced total mesophilic bacterial counts on the lettuce by about 1 log CFU/g over 7 days (relative to control packages) and slowed the development of off-odors. However, some panelists could detect a slight herbal scent in the salad, indicating the need to optimize the dosage. This example reflects a common industrial consideration in finding the balance between efficacy and sensory impact. In this trial, a reduction in TTO amount or improved encapsulation was identified as the next step to make the solution market-ready.

Another real-world example is from the meat-packing industry. A meat processor explored using tea tree oil in the rinse water for poultry carcasses as a natural antimicrobial intervention (to kill *Campylobacter* and *Salmonella* on chicken surfaces). TTO was added at a low level (around 0.2%) to the chiller water. The results were mixed as there was a measurable reduction in bacterial contamination on the chicken (about 0.5 log CFU reduction compared to no TTO), but the processing plant workers reported a strong, camphor-like odor in the chiller area and on the chicken skin [189]. While the odor mostly dissipated after air-chilling, the company was cautious about consumer perception. This highlights the issue of volatile carry-over; even if the product does not retain much smell, the use of fragrant oils in processing can raise questions. Nevertheless, with better ventilation and perhaps by using a less aromatic fraction of TTO (e.g., removing some of the more pungent terpenes), this approach could be refined. The plant noted that tea tree oil did not foam or leave residues in equipment, which is a positive from a sanitation standpoint.

On the commercial product front, there are already retail items leveraging tea tree oil for oral health, which indirectly relates to food safety and hygiene. For example, an antimicrobial chewing gum containing tea tree oil is sold in some markets as a therapeutic gum for oral hygiene. These products often combine a low dose of TTO with other flavors (like mint or green tea) and xylitol [190]. According to the manufacturer, the gum’s essential oil components help reduce oral bacteria that cause plaque and bad breath. While not food, chewing gum is regulated as a food item and demonstrates that TTO can be formulated into an edible matrix that consumers can chew (but generally not swallow) for antimicrobial benefit. This concept could potentially extend to functional foods. For instance, a lozenge or tablet that releases tea tree oil in the mouth to sanitize the throat (though currently TTO’s strong taste and the risk of ingestion have limited such developments) [191]. The chewing gum case also provides a safety precedent, as these gums typically contain only a very small amount of tea tree oil (a few milligrams per piece), such that even if some oil is ingested, it is below harmful levels. Indeed, safety is paramount; regulatory agencies and toxicology studies caution that tea tree oil should not be swallowed in large amounts, as it can be toxic if misused. The oral care products with TTO are designed for local action in the mouth with minimal swallowing. This principle would similarly apply if one were to design a tea tree oil-infused edible film for, say, a deli meat: the dose must be controlled and likely combined with other ingredients to ensure consumer safety.

From an industrial scalability perspective, tea tree oil is readily available in large quantities (it is produced from *Melaleuca* plantations, especially in Australia). It is already used in cosmetics and healthcare products, so supply chains and quality standards (ISO for tea tree oil composition) are well-established. Food-grade tea tree oil would need to meet purity criteria and absence of contaminants (e.g., no heavy metals, pesticides) [192]. Cost-wise, TTO is moderately priced among essential oils, but using it in foods at effective doses could be more expensive than synthetic preservatives. This is where its potent activity is a plus: even at <1% incorporation, it can achieve results that might require higher percentages of other natural extracts.

In the United States, many essential oil components (like terpinene-4-ol, eucalyptol) are approved as flavorings [193]. Tea tree oil itself, while not commonly used as a flavor, could be legally added in small amounts under the flavoring exemption, though not explicitly as a preservative on the label. In the EU, tea tree oil would fall under flavoring or novel food regulations if ingested; currently, it is more likely to be accepted for use in active packaging (which is considered a packaging material, not an ingredient) [194]. Therefore, initial commercialization of TTO for food safety might occur via packaging innovations that do not require labeling the oil on the food product. This strategy has been used for other essential oils, for example, oregano oil sachets in bread packaging. Furthermore, recent developments in organic nanoparticle-based antimicrobial systems have highlighted the potential of nano-encapsulation and active packaging technologies to enhance stability, controlled release, and antimicrobial performance of natural bioactive compounds in food applications, providing a promising pathway for future TTO commercialization [145,195].

In conclusion, the translation of tea tree oil applications from lab to industry is underway but still in early stages. The real-world case studies show both the potential and the hurdles: TTO can clearly improve microbial safety and shelf-life in various foods, yet attention must be paid to sensory effects, consumer acceptance, and regulatory compliance. Continued collaboration between food scientists and industry will likely yield optimized formulations (perhaps blending tea tree oil with other milder essential oils or using ultra-controlled release) that can deliver the food safety benefits without downsides. As clean-label preservation and natural antimicrobials gain traction, tea tree essential oil stands out as a compelling option, one with a long history of antimicrobial use outside of food, now being adapted for keeping our foods safe and fresh.

### 4.8. Oral and Functional Food Applications

Beyond direct food preservation, tea tree oil finds applications in oral health products and could be considered in functional foods that intersect with food safety (for example, products aimed at reducing oral or gastrointestinal pathogens). The antimicrobial properties of TTO have been harnessed in dentistry and oral hygiene, which, while not traditional food safety, relate to controlling microbes in the oral cavity, essentially the first step of the food consumption pathway.

Tea tree oil is a popular ingredient in oral care products like mouthwashes, toothpastes, and gels for its ability to combat oral pathogens. Studies show that TTO is effective against common oral bacteria such as *Streptococcus mutans* (which causes cavities) and *Porphyromonas gingivalis* (involved in gum disease). For instance, a clinical study using a tea tree oil gel in patients with chronic periodontitis found significant reductions in plaque and gingival inflammation compared to placebo [196]. Ripari et al. (2020) compared the clinical effectiveness of a TTO mouthwash with a 0.12% chlorhexidine (CHX) mouthwash over 14 days in 42 adults with plaque-induced gingivitis [197]. Both treatments significantly improved gingival health, but they differed in specific clinical outcomes and side-effect profiles. In the TTO group, plaque index (PI) decreased markedly from 53.25% to 5.50%, and bleeding index (BI) declined from 38.41% to 4.22%, with TTO not causing any dental dyschromia or taste alteration, and only 18% of users reported mild, temporary nausea due to its aroma. In contrast, 20% of CHX users developed dental staining. These results suggest that TTO may serve as a viable, non-toxic alternative for managing gingivitis, particularly for individuals prone to CHX-related side effects or seeking natural oral care options.

TTO likely helps by reducing the microbial load in the mouth, which in turn contributes to safer ingestion of foods since fewer pathogens reside orally to possibly travel into the gut with food. Given TTO’s toxicity if swallowed in large amounts, these oral products are typically used and expectorated. As the Colgate Oral Care Center advises, tea tree oil may help control bacteria and plaque, but it should only be used in products that are spat out, not swallowed [198]. This aligns with general safety guidance that TTO should not be ingested directly due to risk of neurologic effects if consumed at higher doses.

One intriguing product is the antimicrobial chewing gum containing tea tree oil. Marketed as a functional chewing gum for oral health, it combines a small dose of tea tree oil with breath-freshening flavors. Chewing this gum after meals could help reduce oral bacteria and thus prevent halitosis and dental plaque buildup. The gum format slowly releases TTO as the person chews, ensuring prolonged contact with oral surfaces. According to product descriptions, such gums have an antiseptic effect with tea tree oil and green tea, stimulate saliva, neutralize pH, and protect against bacteria and acid attacks on teeth. Essentially, they aim to create a cleaner oral environment, which is beneficial not only for dental health but arguably for overall food safety.

The application of TTO in gastrointestinal-targeted products remains largely hypothetical. Although its antimicrobial constituents exhibit broad-spectrum activity in vitro, oral administration raises important safety concerns because TTO is not intended for routine dietary consumption. Future delivery strategies, such as enteric-coated or controlled-release encapsulation systems, may reduce direct exposure while targeting specific regions of the gastrointestinal tract. However, such approaches require rigorous evaluation of bioavailability, efficacy, and long-term safety before clinical or food applications can be considered. Current evidence suggests that formulation and dosage are the primary determinants of safety. Regulatory agencies have highlighted potential risks associated with oral exposure to tea tree oil, including neurotoxicity and possible genotoxicity at excessive doses [199]. Consequently, any future gastrointestinal or functional food applications should prioritize controlled delivery, minimal effective concentrations, and comprehensive toxicological assessment. At present, TTO appears more suitable for external food preservation and antimicrobial packaging than for direct oral consumption, and further research is needed to establish a safe therapeutic window for ingestible applications.

In terms of consumer acceptability, the oral care applications of tea tree oil have generally found a positive niche. Many people seek natural remedies for gum disease or bad breath and are willing to tolerate a medicinal taste for the sake of efficacy. Nonetheless, products often mask tea tree’s flavor with mint or other strong flavors. For example, a tea tree oil mouthwash might include menthol and herbal extracts to create a more pleasant taste while delivering the antimicrobial punch. Similarly, an oral hygiene candy or gum could blend sweet, minty, and herbal notes.

In conclusion, while not a conventional food preservative use, tea tree essential oil’s role in oral antimicrobial applications complements food safety by reducing the microbial burden at the point of food entry (the mouth). It exemplifies how TTO can be formulated in ingestible or semi-ingestible products to provide health benefits. Any expansion of TTO into functional foods or supplements would require careful formulation to ensure safety. At present, its use in chewing gums and mouthwashes is the main foray in this arena, and these products demonstrate that with low concentrations and proper use instructions, TTO can be a valuable component for maintaining microbial control in the domain of personal and food-related hygiene.

## 5. Challenges, Safety Concerns, and Prospects of TTO in Food Safety Systems

Tea tree essential oil (TTO) exhibits potent antimicrobial properties and has been explored as a natural preservative in food applications. However, translating its efficacy from laboratory studies to real-world food systems faces numerous challenges. This section reviews the key safety concerns and technical hurdles limiting TTO’s use in food safety (Table 8), as well as regulatory constraints and prospects for future innovations. The discussion is organized into subsections on toxicity and safety, sensory impacts, regulatory landscape, technical challenges, and future directions.

### 5.1. Toxicity and Safety Concerns

The toxicological profile of TTO imposes fundamental constraints on its use in food systems and distinguishes it categorically from conventional culinary essential oils. TTO has no history of intentional ingestion and is classified as an oral toxin, with even small ingested quantities associated with central nervous system depression, ataxia, and aspiration pneumonia [201]. At the cellular level, TTO constituents exhibit cytotoxicity toward mammalian cells at concentrations overlapping with antimicrobial-effective doses; in vitro cell viability is significantly reduced above approximately 0.1%, establishing a narrow safety margin that complicates dose optimization for food applications [26,202]. Dermal exposure carries additional risks, including allergic contact dermatitis in 1–4% of dermatological patients, a hazard amplified by oxidative degradation products such as peroxides and ascaridole that accumulate under improper storage conditions.

Chronic toxicity further restricts translational applicability. Terpinen-4-ol has demonstrated testicular toxicity in rats, forming the basis for the EU’s classification of TTO as a presumed reproductive toxin [203]. Trace quantities of methyl eugenol, a genotoxic and carcinogenic compound in animal models, reinforce the need for strict migration limits in any food-contact application. Evidence of weak estrogenic and anti-androgenic activity among TTO constituents has prompted precautionary guidance against use in pediatric and perinatal populations, though a causal link to human endocrine pathology remains unestablished. It is recommended that nanoencapsulation may reduce acute irritation by preventing burst release, but nanoscale delivery may also increase cellular uptake and alter biodistribution. Therefore, toxicological evaluation should consider both the intrinsic toxicity of TTO and carrier-dependent effects.

These findings collectively constrain viable deployment strategies to indirect-contact configurations, including active packaging, antimicrobial surface coatings, and encapsulated systems engineered for minimal terpene migration, supported by rigorous food-matrix-specific safety validation and transparent allergen labeling.

### 5.2. Sensory and Organoleptic Impacts on Food

Another major limitation of TTO in food applications is its strong sensory profile. TTO exhibits a characteristic camphoraceous, medicinal aroma and a bitter, eucalyptus-like taste, which can be incongruent with most food products. Even at low concentrations, volatile terpenes such as terpinene, cineole, and terpineol impart noticeable flavors, raising significant organoleptic concerns.

Studies show that essential oils typically require concentrations of 0.1–1.0% to achieve antimicrobial efficacy in foods, yet these levels often exceed sensory acceptance thresholds. For example, thyme oil at 0.9%, although effective against pathogens, rendered meat products unacceptable to consumers [204]. Similarly, TTO at concentrations as low as 0.1% (*v*/*v*) can already introduce a detectable medicinal flavor, while bactericidal levels (0.5–1.5%) are likely to overwhelm natural food characteristics [7].

Unlike commonly accepted flavoring oils (e.g., basil or citrus), TTO lacks culinary familiarity, making its sensory impact more difficult to mask. Empirical studies on meat and seafood systems have reported extended shelf life with TTO treatment but also noted the development of off-odors and reduced sensory scores, highlighting the trade-off between antimicrobial efficacy and consumer acceptance [16].

Mitigation strategies include dose optimization, encapsulation to delay aroma release, and pairing with strong or compatible flavors. However, these approaches offer limited success, as even minor residual TTO can alter perceived freshness and quality. Consumer studies consistently indicate that essential-oil-treated products often score lower in flavor and overall acceptability, with descriptors such as “medicinal” or “bitter” frequently reported [69].

### 5.3. Regulatory Landscape

The regulatory landscape governing TTO in food systems presents a substantial barrier to its direct application and reflects the unresolved toxicological concerns outlined above. In the United States, TTO lacks Generally Recognized as Safe (GRAS) status and is absent from FDA-authorized food additive lists; while FEMA has assigned TTO an evaluative number as a flavoring substance, this carries no official regulatory clearance, and no food additive petition has been pursued by manufacturers. In the European Union, TTO is similarly excluded from EFSA’s list of authorized flavoring preparations, and national-level assessments have been largely prohibitive. A 2020 ANSES risk opinion concluded that oral ingestion of *Melaleuca* oils poses neurological, genotoxic, and reproductive hazards, effectively precluding their use in French food and supplement products and signaling broader EU-level skepticism [199]. At the international level, neither Codex Alimentarius nor JECFA has established provisions or safety specifications for TTO as a food additive. The most advanced regulatory recognition to date remains EFSA’s 2024 authorization of TTO as a sensory feed additive under Commission Implementing Regulation (EU) 2025/1402, a decision explicitly limited to animal feed at tightly controlled inclusion levels and not extended to human food systems [205].

For researchers and industry stakeholders seeking to deploy TTO in food safety applications, this regulatory environment defines clear operational boundaries. Viable pathways are limited to configurations that minimize direct human exposure, such as active packaging systems engineered for negligible terpene migration or post-harvest surface treatments with verified removal efficacy, and must be supported by comprehensive safety dossiers encompassing migration testing, realistic consumer exposure modeling, and evaluation of potentially genotoxic trace constituents such as methyl eugenol. Substantive progress toward regulatory acceptance will require sustained toxicological investigation and proactive engagement with competent authorities. Future nano-enabled TTO formulations should adopt safe-by-design principles through the careful selection of food-grade carrier materials, minimization of nanoparticle loading, optimization of release kinetics to avoid excessive exposure, and systematic evaluation of migration, gastrointestinal fate, chronic toxicity, and environmental persistence. A safe-by-design framework may therefore provide a practical pathway for translating nano-enabled TTO formulations from laboratory-scale studies to commercially viable food preservation systems by balancing antimicrobial performance, consumer safety, regulatory compliance, and environmental sustainability.

### 5.4. Technical Challenges in Food Applications

**Figure 9:**
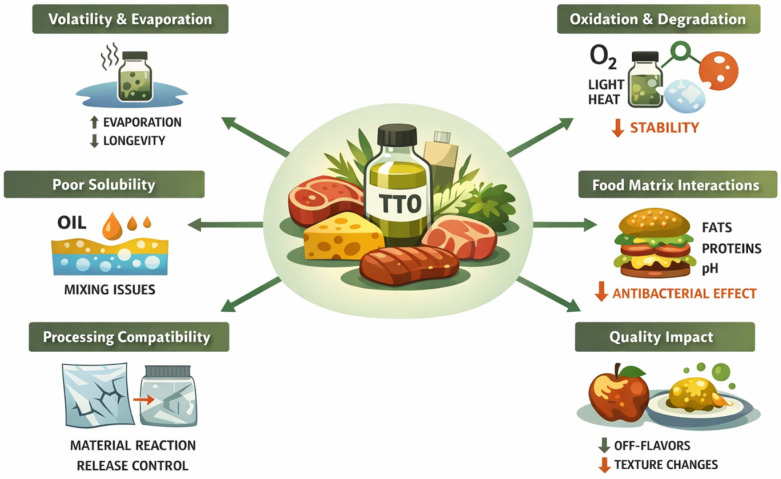
Technical challenges limiting the application of TTO in food systems (created by BioRender and modified by Figure Labs).

Beyond toxicity and legal status, a suite of technical challenges arises when deploying TTO in real food systems. Essential oils, including TTO, behave differently in complex foods than in simple lab media. Key issues include volatility and stability, solubility and dispersal in the food matrix, interactions with food components, and maintaining efficacy over a product’s shelf life (Figure 9).

#### 5.4.1. Volatility and Evaporation

TTO is an exceedingly volatile oil, a characteristic advantageous for aromatherapy but detrimental for prolonged antibacterial efficacy. Upon application to a surface or integration into packaging, TTO tends to evaporate rapidly due to its low-boiling components. Research indicates that most applied essential oils will evaporate within hours at room or slightly higher temperatures [206]. One investigation indicated that approximately 84% of a TTO sample evaporated from the surface within 2 h at 30 °C, with complete evaporation occurring within 8 h [207]. This indicates that in a culinary environment, TTO may evaporate before fully delivering its antibacterial properties, particularly when the product is subjected to air or mild heat. Elevated volatility indicates that oil cannot be effectively contained within packaging materials without specialized encapsulation, as it will migrate into the headspace or atmosphere. Therefore, administering a sustained effective dose of TTO in a food product or processing environment is difficult; frequent reapplication or controlled-release methods are necessary to mitigate fast evaporation. Volatility is also related to sensory impact, with a volatile oil rapidly saturates a container’s headspace with odor, potentially notifying customers, which may be undesirable for active packaging if it indicates the presence of chemicals.

#### 5.4.2. Oxidation and Chemical Stability

TTO’s components (terpenes like terpinene, terpineol, etc.) are prone to oxidation when exposed to oxygen, light, or heat [21]. Over time, an essential oil can degrade, losing its antimicrobial potency and forming new compounds. In TTO, oxidation products (e.g., peroxides, ascaridole) not only risk allergenicity but may also have different odors (often harsher or rancid notes) that could further spoil food aroma. For practical use, the oil would need protection from oxidation. Adding antioxidants or using protective packaging, adding antioxidants must be food-approved, and they might not fully halt oxidation if the oil is exposed to air [208]. Additionally, the antimicrobial efficacy of TTO generally comes from its unoxidized constituents; as those degrade, the efficacy could drop. Therefore, maintaining the shelf stability of TTO formulations is a technical hurdle. Any TTO-treated product would require validation of how long the oil remains active and in what form. Refrigeration and darkness can extend stability, but many foods experience fluctuating conditions.

#### 5.4.3. Poor Water Solubility and Dispersion

TTO is hydrophobic and essentially insoluble in water [16]. Foods, especially aqueous or high-moisture foods, pose a problem for incorporating such oil. Uniform dispersion of TTO in a food matrix is difficult without an emulsifying system. If one simply adds TTO to a water-based food (soup, beverage, brine, etc.), it will float or settle out, leading to uneven distribution and possibly localized high concentrations (hotspots) that could be toxic or cause flavor bursts. Even in lipid-containing foods, the oil may preferentially partition into fat phases, not necessarily where microbes reside. Many studies note that EOs can be effectively antimicrobial in vitro, but in actual foods, their efficacy diminishes due to distribution issues [209]. The presence of emulsifiers, surfactants, or encapsulation systems is often required to disperse essential oils uniformly. For instance, creating an oil-in-water emulsion or nanoemulsion of TTO can help solve this, but this adds complexity and potential cost. Process compatibility is also a factor; if a food is liquid, one might disperse TTO with high-shear mixing or ultrasonication, but in solid foods (meat, fruits) one might need to coat the surface or use a packaging approach.

#### 5.4.4. Interactions with Food Matrices

The matrix effect is a well-documented challenge for essential oils. Components of foods (fats, proteins, carbohydrates, salt, pH, etc.) can interact with or absorb essential oils, reducing the free concentration available to act on microbes. For example, high-fat foods like cheese or sausage can sequester lipophilic compounds such as terpene alcohols within the fat phase [210]. This not only lowers antimicrobial efficacy (since less oil is in the aqueous phase where bacteria may be active) but can also protect microbes by trapping the oil away from them. Proteins can bind flavor compounds and might bind some terpenes as well, further diminishing activity. Studies have found that essential oils are typically more effective in simple media or low-fat foods than in rich, high-protein/fat foods [65]. Additionally, pH can influence the antimicrobial action of oils, though TTO’s constituents are not ionizable acids like some preservatives; extreme pH conditions might affect the integrity or solubility of the oil. Food microstructure also matters in a solid or semi-solid food, diffusion of TTO may be limited. The oil might act only near the surface, failing to reach microbes in the interior. All these factors mean that achieving reliable microbial inhibition with TTO in a complex food often requires a higher initial dose than in a laboratory broth, which circles back to the sensory and safety issues of using a higher dose.

#### 5.4.5. Compatibility with Processing and Packaging Materials

If TTO is applied as part of a packaging system or coating, its chemical nature can pose challenges. It may interact with packaging polymers. For instance, terpenes can swell or diffuse into certain plastics (like polyethylene or polystyrene), potentially weakening the material or causing loss of the oil. Some active packaging research encapsulates EOs in sachets or coatings to avoid direct contact with polymers [211]. There is also the issue of controlled release; ideal active packaging would release TTO slowly over time to prolong antimicrobial action, but engineering such release is complex. TTO’s vapor pressure means it tends to evaporate quickly unless trapped in a carrier matrix. Achieving a sustained, low-level release often requires special carriers (e.g., cyclodextrins, porous solids, liposomes) which must be food-safe and stable. Additionally, from a manufacturing perspective, incorporating volatile oils like TTO into packaging might require modifying production processes (for example, extrusion temperatures would need to be low enough not to drive off the oil, or coating methods must ensure even distribution) [212].

#### 5.4.6. Effect on Food Quality Parameters

In addition to flavor, TTO may influence other quality characteristics. Essential oils may demonstrate antioxidant action, potentially advantageous in preventing lipid oxidation in food products. TTO exhibits documented antioxidant capacity in specific experiments. Nevertheless, if improperly prepared, the oil may result in discoloration or surface residues on food items. Consumers may perceive oily films or droplets on food surfaces as deterioration or adulteration [213]. Furthermore, elevated quantities of TTO may interact with dietary constituents. For example, terpenes may participate in acid-catalyzed processes in acidic foods or polymerize under specific conditions, thereby modifying texture or appearance. These features are rarely examined; however, any extensive application must guarantee that food quality (color, texture, shelf-life) remains unaffected by the incorporation of TTO or its chemical alterations during storage [214].

In summary, the technical hurdles in applying TTO for food safety are non-trivial. Keeping the oil where it is needed, in an active form, at an effective concentration, and uniformly distributed, for the duration of the product’s shelf life, without harming the food’s integrity, is a multifaceted engineering problem. Traditional methods of adding preservatives, simply mixing them into the formula, do not work well with essential oils like TTO. These challenges have spurred research into advanced delivery systems and formulation technologies. Overcoming these technical barriers is key to unlocking TTO’s potential as a natural preservative while minimizing its downsides.

### 5.5. Future Directions and Prospects

Despite the challenges outlined, TTO remains of great interest as a natural antimicrobial. Researchers are actively exploring innovative solutions to harness TTO’s benefits in food safety while addressing safety, sensory, and stability issues. Future directions include advanced formulation technologies, synergistic hurdle approaches, intelligent packaging systems, and sustainability-driven strategies (Table 9).

#### 5.5.1. Novel Formulation and Delivery Systems

One of the most promising strategies is to reformulate TTO into forms that mitigate its volatility and sensory impact. Nanoemulsions and encapsulations can dramatically improve the dispersion and stability of essential oils. By creating nanoscale oil droplets (20–200 nm) with food-grade emulsifiers, TTO can be effectively dissolved in aqueous systems, yielding a homogeneous distribution and controlled release. Nanoemulsified essential oils often show enhanced antimicrobial efficacy at lower doses and can be less perceptible in taste. For example, formulating TTO into a fine emulsion or liposomal system might allow it to interact with bacterial cells more readily while using a smaller total quantity of oil [219]. Additionally, encapsulation in biopolymers (such as polysaccharide or protein matrices) or inclusion complexes (e.g., β-cyclodextrin inclusion complexes) can protect TTO from oxidation and modulate its release. Cyclodextrins have been successful in encapsulating volatile compounds. β-Cyclodextrin is approved as a food additive and can trap TTO molecules in its hydrophobic cavity, preventing immediate evaporation. This not only prolongs antimicrobial activity but also reduces the instantaneous odor intensity. Research has demonstrated that cyclodextrin-encapsulated essential oils release more slowly and maintain effectiveness during storage. Another cutting-edge development is loading TTO into structured carrier systems like mesoporous silica or covalent organic frameworks. For instance, a recent study incorporated TTO into a novel covalent organic framework (a porous nanostructured material) and then into a starch-based film, achieving pH-triggered slow release of TTO and significantly improved shelf-life of blueberries [156]. These kinds of systems exemplify how material science innovations can overcome TTO’s instability by anchoring the oil in a matrix until needed. Overall, future formulation work aims to create stealth forms of TTO, wherein the oil is present and active against microbes, but its volatility and flavor are tamed by encapsulation or binding.

#### 5.5.2. Synergistic Hurdle Approaches

The future of natural preservatives likely lies in combination strategies, where multiple mild hurdles work together rather than relying on a single high-dose additive. For TTO, this means using it in synergy with other antimicrobials or processes to achieve the desired microbial control with lower concentrations. Synergistic essential oil blends are one option. Studies have found that combining different essential oils or key constituents can produce a greater-than-additive effect against microbes. In the case of TTO, blending it with oils that have complementary antimicrobial spectra (such as oregano, thyme, or clove oil, which are rich in phenolic compounds) might allow a reduced amount of each oil, thereby diluting individual strong flavors while maintaining efficacy [220]. There is evidence that terpinen-4-ol (tea tree’s main component) can synergize with phenolics like carvacrol or eugenol, perhaps by attacking microbes via multiple mechanisms. Some commercial products already exploit essential oil mixtures for preservative effects, and future research can pinpoint optimal ratios that minimize sensory impact. Beyond other oils, natural antimicrobial compounds like nisin (a bacteriocin), organic acids, or plant extracts could be combined with TTO. For example, a small amount of TTO might greatly enhance the effectiveness of fermentation or vinegar against *Listeria*, allowing each to be used at sub-threshold levels [221].

Another synergy approach involves pairing TTO with emerging preservation technologies. This is aligned with the hurdle technology concept in food safety. Non-thermal interventions such as mild heating, high-pressure processing (HPP), ultrasound, pulsed light, or cold plasma can stress or damage microbes just enough that they become more susceptible to antimicrobial agents. Research has shown that applying essential oils in conjunction with HPP or pulsed electric fields, for instance, yields a synergistic kill, enabling a lower dose of oil than would be needed alone. In one study, combining a moderate HPP treatment with a low concentration of EO achieved the same microbial reduction that required a much higher EO dose without HPP [222]. Similarly, a slight increase in temperature, but still below cooking levels, can potentiate the effect of TTO, since heat can make bacterial cell membranes more permeable to the oil’s components. The future might see integrated preservation processes with a chilled pasteurization step in a TTO-infused marinade for meat, resulting in extended shelf life with minimal sensory change. By reducing the required concentration of tea tree oil through such synergies, both safety and taste concerns are alleviated. This approach requires interdisciplinary planning to ensure the combined hurdles do not adversely affect food quality, but it is a promising way to leverage TTO’s strengths efficiently.

#### 5.5.3. Active and Smart Packaging Systems

TTO’s high volatility, while troublesome for direct addition, can be advantageous in active packaging applications. The idea is to incorporate TTO into packaging materials or inserts that slowly release the oil vapor into the headspace of the food package, thus inhibiting microbial growth on surfaces and in air contact without saturating the food with oil [223]. This approach can significantly improve the shelf-life of perishable goods (e.g., fresh produce, meats) by creating an antimicrobial atmosphere. Recent advances include encapsulated essential oil sachets or coatings within packages. For instance, researchers have developed β-cyclodextrin-based coating on cardboard or plastic that contains TTO; the oil remains trapped until it gradually volatilizes, providing sustained antimicrobial action during storage [224]. Such packaging can be considered smart if it responds to environmental triggers, as some systems aim to release more oil when humidity or temperature rises (conditions that often coincide with microbial risk). In the earlier example of TTO in a covalent organic framework within a starch film, the release was pH-sensitive, which could be tuned to respond to food spoilage conditions, where spoilage can increase pH in some cases.

**Figure 10:**
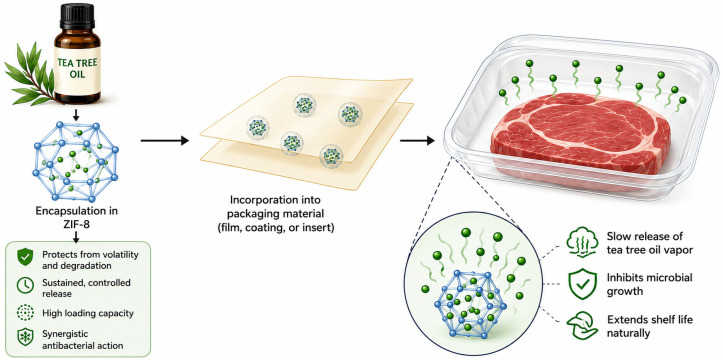
Schematic illustration of TTO encapsulation within the ZIF-8 metal–organic framework, followed by incorporation into food packaging materials (films, coatings, or inserts) (created by BioRender and modified by Figure Labs). The porous ZIF-8 structure protects volatile TTO components from degradation and enables sustained vapor release during storage. The gradual release of TTO inhibits microbial growth on food surfaces, thereby extending shelf life while reducing the amount of essential oil required.

Encapsulating TTO within metal–organic frameworks, such as ZIF-8, offers a potentially synergistic strategy to enhance the antibacterial performance of both components. The porous structure, high surface area, and tunable chemistry of ZIF-8 enable efficient loading of hydrophobic essential oils’ constituents while protecting them from volatility and oxidative degradation. This encapsulation promotes sustained and controlled release, improving contact time with microbial cells [225]. Simultaneously, the intrinsic antibacterial activity of ZIF-8, often associated with Zn^2+^ ion release and reactive oxygen species generation, can act in concert with TTO’s membrane-disruptive terpenes, leading to multi-modal antimicrobial action. Such combined mechanisms enhance permeability, destabilize cellular homeostasis, and reduce the likelihood of resistance development (Figure 10). As a result, TTO encapsulated ZIF-8 systems show strong potential as advanced antimicrobial platforms for food safety, particularly in coatings, packaging, and surface sanitation applications.

The benefit of active packaging is that the consumer may not ingest the oil at all; it mostly acts in the package headspace or on the surface, and residual amounts tend to dissipate when the package is opened. This could largely circumvent regulatory barriers since TTO is not being added to the food but rather used in packaging material, which, if compliant with food contact regulations, is permissible.

Future smart packaging might integrate time–temperature indicators or sensors that could trigger release of TTO when conditions warrant (e.g., if refrigeration fails or if microbial counts begin to rise). While such sophisticated systems are still conceptual, the combination of smart controlled-release technology with natural antimicrobials like TTO is a compelling direction for ensuring food safety in supply chains. Importantly, these packaging solutions also resonate with consumer desires for clean-label preservation, and the packaging can be marketed as an innovative, chemical-free safety feature.

#### 5.5.4. Sustainability and Natural Sourcing Considerations

The increasing interest in TTO for food preservation reflects a broader shift toward renewable, bio-based antimicrobial technologies. Derived from the leaves of *Melaleuca alternifolia*, TTO is produced from a renewable plant resource that can be cultivated using relatively low-input agricultural practices [224]. However, the sustainability of TTO should be evaluated across its entire life cycle rather than based solely on its natural origin. Life cycle assessment (LCA) studies indicate that extraction is a major contributor to the environmental footprint of essential oil production. For example, supercritical CO_2_ extraction has been reported to require substantially less energy and generate lower greenhouse gas emissions than conventional steam distillation [226]. Consequently, future studies should compare cultivation, extraction, nanoformulation, packaging manufacture, and end-of-life disposal to determine the overall environmental performance of TTO-based preservation systems.

Nanoencapsulation may further improve sustainability by reducing volatilization, protecting bioactive constituents from degradation, and enabling controlled release, thereby lowering the amount of essential oil required to achieve antimicrobial efficacy. When incorporated into biodegradable packaging materials, encapsulated TTO may also reduce reliance on synthetic preservatives while contributing to shelf-life extension and food waste reduction [227]. Nevertheless, these potential environmental benefits remain largely hypothetical because comparative LCA and techno-economic analyses of nano-enabled TTO packaging are still limited. Future work should therefore integrate antimicrobial performance with environmental, economic, and industrial feasibility assessments.

Despite its promising antimicrobial properties, successful translation of TTO into commercial food systems will depend on addressing several remaining challenges, including sensory acceptability, standardized migration testing, long-term safety evaluation, and regulatory approval. Future research should adopt interdisciplinary approaches that combine material science, food microbiology, toxicology, sensory science, and packaging engineering to optimize controlled-release formulations while ensuring consumer safety and product quality. Rather than focusing solely on antimicrobial efficacy, next-generation TTO delivery systems should be evaluated using comprehensive performance metrics that balance effectiveness, sustainability, regulatory compliance, and commercial feasibility.

## 6. Outcomes and Focus Areas for TTO’s Future Use

TTO-based treatments have demonstrated meaningful antimicrobial performance in food applications, with multi-log reductions in target pathogens and shelf-life extensions ranging from several days to more than one week across diverse food systems [167,228]. However, sensory performance remains highly dependent on formulation, dosage, and food matrix. Foods with strong intrinsic flavors or those subjected to thermal processing, such as meat, poultry, and seasoned seafood, generally tolerate residual terpene notes more readily than delicate products such as fresh dairy, where antimicrobial-effective concentrations may negatively affect flavor. Nevertheless, several studies have reported that optimized formulations, particularly those employing encapsulation or controlled-release systems, maintained antimicrobial efficacy without significantly altering consumer sensory perception. In some cases, treated products even exhibited improved odor quality due to suppression of spoilage-associated volatile compounds.

The regulatory status of TTO in food systems continues to depend on both jurisdiction and intended application. In the United States, several essential oils from edible plants are recognized as generally recognized as safe (GRAS) for flavoring purposes, although TTO has not been specifically approved as a food preservative. Similarly, European regulations permit certain essential oils within established safety limits while requiring evidence that intended use does not compromise consumer health [229]. Consequently, future commercialization of TTO-based preservation systems will require comprehensive toxicological evaluation, migration assessment for packaging applications, and compliance with food additive or food-contact material regulations, together with appropriate product labeling where preservation is the primary technological function [230].

Current evidence suggests that TTO has the greatest translational potential in high-moisture food systems, including meat, poultry, seafood, fresh produce, and fruits susceptible to fungal spoilage. Its application is comparatively less suitable for mild-flavored dairy products, where sensory constraints limit effective concentrations, and for low-moisture foods in which rapid volatilization reduces antimicrobial persistence. Continued progress will therefore rely on formulation strategies that enhance stability while minimizing sensory impact, including nanoencapsulation, metal-organic framework carriers, edible coatings, and synergistic combinations with other natural antimicrobials.

Looking forward, data-driven formulation design may further accelerate the development of TTO-based preservation systems [231]. Rather than relying solely on empirical trial-and-error approaches, statistical modeling and machine learning could be used to identify optimal combinations of TTO concentration, encapsulation materials, release profiles, food matrices, storage conditions, and sensory constraints. Such predictive approaches may reduce experimental workload while improving formulation efficiency and facilitating translation from laboratory-scale studies to commercial food applications. Combined with pilot-scale validation and regulatory approval, these advances could position TTO as a practical component of next-generation clean-label antimicrobial technologies.

## 7. Conclusions

Tea tree essential oil (TTO), primarily derived from *Melaleuca alternifolia*, represents a promising natural antimicrobial for food safety applications. This review highlights its physicochemical characteristics, broad-spectrum antimicrobial mechanisms, and emerging roles across diverse food systems. The bioactivity of TTO is largely attributed to its terpene-rich composition, particularly terpinen-4-ol, α-terpineol, and γ-terpinene, which exert antimicrobial effects by disrupting membranes, inducing oxidative stress, and interfering with quorum sensing and biofilm formation.

Compared with other essential oils such as oregano, thyme, and cinnamon, TTO demonstrates competitive and broad-spectrum efficacy against key foodborne pathogens, including *Listeria monocytogenes*, *Salmonella enterica*, *Escherichia coli*, and *Staphylococcus aureus*, as well as spoilage fungi. Importantly, advances in nano-enabled delivery systems, including nanoemulsions, encapsulation, edible coatings, and active packaging, have significantly enhanced their stability, bioavailability, and antimicrobial efficiency while mitigating volatility and sensory limitations.

Despite these advantages, challenges remain, including strong aroma, potential cytotoxicity at high concentrations, oxidative instability, and variability in regulatory acceptance. Interactions with complex food matrices and consumer acceptance issues further complicate its practical application.

A key limitation in current research is the disconnect between antimicrobial efficacy and practical safety thresholds. Future work must integrate dose–response relationships, toxicological validation, and regulatory compliance to define application-specific safe usage levels, particularly distinguishing between direct food incorporation and indirect exposure via packaging systems. In addition, standardization of composition, safety validation, and regulatory harmonization will be critical for industrial translation. Overall, TTO holds substantial potential as a clean-label, sustainable alternative to synthetic preservatives, particularly when supported by advanced delivery systems and interdisciplinary innovation.

## Figures and Tables

**Table 1:** Major scientific motivations for using tea tree essential oil in modern food safety systems [1,2].

Food Safety Challenge	Applications of TTO	Role of TTO
Increasing antimicrobial resistance	Need for non-antibiotic interventions	Broad-spectrum activity with Gram+/Gram− and fungi
Consumer push for clean-label preservatives	Replacement of synthetic chemicals	Not approved as GRAS for direct food use in the United States, and application depends on the regulatory framework
Food-contact surface contamination	Biofilm persistence in industry	Strong antibiofilm disruption with mitigation of persistent biofilms in processing environments
Limited efficiency of washing/sanitizing	Chlorine/treatments insufficient	Vapor-phase and contact inactivation

**Table 2:** Key physicochemical parameters of tea tree essential oil [31].

Property	Typical Value	Implication for Food Systems
Density	0.885–0.906 g/mL	Floats on water and requires emulsification
Aqueous solubility	~0.03% (300 mg/L)	Poor dispersion without formulation
Vapor pressure	~2.1 kPa at 25 °C	High volatility → suitable for active packaging
Flash point	56–60 °C	Must be handled carefully in processing
Dominant component	Terpinen-4-ol (35–48%)	Major contributor to antimicrobial action

**Table 3:** Functional comparison of major TTO components [33].

Component	Relative Abundance	Functional Group	Contribution
Terpinen-4-ol	Highest	Terpenoid alcohol	Antibacterial, antibiofilm
γ-Terpinene	Moderate–high	Monoterpene	Oxidation-prone; aromatic changes
α-Terpinene	Moderate	Monoterpene	Supports membrane fluidization
1,8-Cineole	1–15%	Oxide	Aroma intensity, low antimicrobial

**Table 4:** Relationship between major tea tree oil constituents and nano-enabled formulation strategies [7,28].

Major Constituent	Physicochemical Characteristics	Formulation Challenge	Nano-Enabled Strategy	Expected Benefit
Terpinen-4-ol	Oxygenated monoterpene, moderate lipophilicity, principal antimicrobial compound	Partial volatilization and oxidation	Nanoemulsions, liposomes, SLNs	Improved stability, sustained release, enhanced antimicrobial efficacy
γ-Terpinene	Highly hydrophobic hydrocarbon, oxidation-sensitive	Rapid oxidation and poor aqueous dispersion	Lipid nanoparticles, polymeric nanoparticles	Reduced oxidation and improved dispersion
α-Terpinene	Hydrophobic monoterpene, membrane-active	Volatility and oxidative degradation	Nanoemulsions, cyclodextrin inclusion complexes	Improved retention and controlled release
1,8-Cineole	Relatively volatile oxygenated terpene	Rapid evaporation and aroma loss	Cyclodextrin complexes, liposomes	Reduced volatility and prolonged release

**Table 5:** Representative MIC/MBC values of TTO against foodborne pathogens [7,94,95].

Microorganism	MIC (% *v*/*v*)	MBC (% *v*/*v*)	Sensitivity
*S. aureus*	0.25–0.50	0.50–1.0	High
*L. monocytogenes*	0.05–0.25	0.25–0.50	Very high
*E. coli* O157:H7	0.20–0.40	0.40–1.0	Moderate
*Salmonella* spp.	0.20–0.80	0.50–1.5	Moderate
*Candida albicans*	0.06–0.50	0.25–1.0	High

**Table 6:** Selected applications of tea tree essential oil in food preservation (2010–2025).

Food Product & Microbial Issue	TTO Application & Formulation	Outcomes (Microbial Control & Shelf-life)	Sensory & Feasibility
Raw chicken fillets—general spoilage (bacteria, oxidation)	1% TTO in marinade/dipping solution (lab-scale trial)	Decreased total viable counts; slowed spoilage, +7 days refrigerated shelf-life vs. control. Also decreased lipid oxidation (TBARS) over 9 days.	Maintained color and odor better than control (fewer off odors). Concluded as an effective natural preservative for meat [62].
Raw chicken meat—Salmonella contamination	Chitosan nanofiber mat with TTO-loaded liposomes (active packaging)	~5 log reduction in Salmonella on chicken within 4 days at 12–25 °C; prevented microbial recontamination, extending safety and shelf-life.	Minimal flavor impact: TTO nanofiber caused no noticeable sensory change in chicken. Demonstrated practicability for pathogen control [108].
Fresh lettuce (Butterhead)—field microflora & coliforms	Preharvest spray with TTO emulsion (single or repeated applications late in growth)	Decreased native mesophilic bacteria and coliforms at harvest and after storage. After 5 days @5 °C, treated lettuce had ~2 log_10_ lower total counts vs. untreated.	No significant differences in sensory quality (appearance, taste) vs. control after treatment. TTO did not adversely affect lettuce flavor [109].
Soft cheese (Feta, fresh Mozzarella)—*Listeria*, *E. coli* risk	Direct EO addition to cheese or brine (0.5–1% needed for activity)	High TTO concentrations in vitro inhibit *L. monocytogenes* and *E. coli*; however, efficacy drops in high-fat cheese. Thyme or clove EO often outperforms TTO against Listeria.	Sensory hurdle: 1% TTO imparted strong off flavors in Feta; panelists “disliked” TTO aroma in Fior di Latte cheese. Thus, TTO’s use in cheese is limited by flavor at effective doses [110].
Strawberries—postharvest spoilage (fungi, quality loss)	β-cyclodextrin/nano-clay microcapsules releasing TTO in package	TTO vapor slowed decay: treated berries stayed mold-free and firm ~3–6 days longer than control at 4 °C. Optimal dose (5 g microcapsules per 1.2 L) ⇒ least decay, lower weight loss, delayed ripening indices.	Maintained fruit appearance and nutrients better during storage. Controlled-release microcapsules prevented overpowering odor; berries’ aroma remained acceptable (no TTO off-taste noted) [111].
Banana—anthracnose (fungal rot by *Colletotrichum*)	Edible coating/film: bilayer sodium alginate film with TTO nanoemulsion + TiO_2_ nanoparticles	Markedly suppressed anthracnose lesions. 3 µg/mL TTO in coating reduced rot severity and extended banana shelf-life; treated fruit had significantly less decay over 12–16 days vs. controls.	No significant sensory detriment reported. The alginate–TiO_2_ matrix slowed TTO release and blocked UV light, preserving fruit quality (firmness, color). Coating is food-grade and meets packaging safety norms [112].
Fresh salmon fillets—spoilage bacteria & oxidation	Electrospun chitosan nanofiber wrapped with encapsulated TTO (coating pad in package)	Lowered microbial loads (including *Listeria*, *E. coli*, *S. aureus* in tests) and slowed spoilage in cold storage. One study showed such EO nanofiber mats added ~6–7 days of shelf-life to fresh fish vs. normal ice storage.	TTO nanofiber inhibited fishy odor development; treated fillets maintained acceptable sensory quality longer. Active fiber is biodegradable (chitosan) and poses no direct residue on fish flesh [113].
Bread (sliced)—mold spoilage (*Penicillium* spp.)	Vapor-phase TTO in package headspace (experimental set-up)	Limited efficacy: TTO vapor only weakly inhibited *P. citrinum* and *P. crustosum* on bread; no meaningful delay of mold growth. (*P. expansum* even grew faster with TTO present).	Bread absorbed some TTO aroma, but doses high enough to suppress molds would likely cause off-flavors. TTO vapor alone is not effective for bread preservation. Other EOs (e.g., lemongrass, clove) show stronger antifungal effects in bakery products [114].

**Table 7:** Summary of the antibiofilm activity of TTO against major foodborne organisms.

Organism	% Biofilm Reduction	Concentration Used	TTO Effectiveness
*Staphylococcus aureus*	50–90%	0.25–1%	Strong membrane disruption [11]
*E. coli*	20–60%	0.5–1%	Strain-dependent [11]
*Pseudomonas aeruginosa*	10–40%	1–2%	Highly resistant strain [133]
*Candida albicans*	40–80%	0.5–1%	Matrix penetration effective [134]

**Table 8:** Advantages and limitations of TTO in food applications [7,69,200].

Advantages	Limitations
Broad antimicrobial spectrum	Strong odor, flavor
Natural, clean label	Limited GRAS approval depending on the country
Works in vapor and liquid phases	Highly volatile, oxidizes quickly
Compatible with nanocarriers	Difficult to disperse in water

**Table 9:** High-potential future research directions.

Innovation	Research Need	Expected Benefit
Smart active packaging	pH/humidity-triggered release	Preventing spoilage events [215]
Synergy blends	Nisin, carvacrol	Lower dose + better taste [216]
Biopolymer–TTO composites	Stability optimization	GRAS-compliant packaging [217]
Microfluidic nanoencapsulation	Narrow droplet size	Better antimicrobial delivery [218]

## Data Availability

No new data were created or analyzed in this study. Data sharing is not applicable to this article.
